# Multi-stakeholder perspective on community pharmacy services in Saudi Arabia: A systematic review and meta-analyses for 2010–2020

**DOI:** 10.1016/j.rcsop.2025.100608

**Published:** 2025-04-28

**Authors:** Khalid S. Alghamdi, Max Petzold, Mahdi H. Alsugoor, Hafiz A. Makeen, Christina Ljungberg Persson, Laith Hussain-Alkhateeb

**Affiliations:** a School of Public Health and Community Medicine, Institute of Medicine, Sahlgrenska Academy, Gothenburg University, Box 469, 40530 Göteborg, Sweden; b Department of Clinical Pharmacy, Faculty of Pharmacy, Al Baha University, Al Baha, Saudi Arabia; c Department of Emergency Medical Services, Faculty of Health Sciences, Al-Qunfudah, Umm Al-Qura University, 21912 Makkah, Saudi Arabia; d Department of Clinical Pharmacy, Faculty of Pharmacy, Jazan University, 6809 Jazan, Saudi Arabia; e Department of Population Health, King Abdullah International Medical Research Centre, King Saud Bin Abdulaziz University for Health Sciences, Riyadh, Saudi Arabia

**Keywords:** Community pharmacy, Pharmacy practice, Services, Barriers, Saudi Arabia

## Abstract

**Background:**

Community pharmacists are the third-largest group of healthcare professionals globally; their capacity often extends far beyond dispensing medication, and they are increasingly involved in a diverse range of advanced health service delivery, which can potentially improve public health outcomes. Among the many initiatives of the Saudi national transformation program towards Saudi Vision 2030 are plans to transform the current model of community pharmacy services by expanding their role to include patient-centered healthcare provision.

**Objectives:**

This systematic literature review (2010−2020) of the multi-stakeholder perspective aimed to evaluate services provided by Saudi community pharmacies and satisfaction levels, investigate perceived factors associated with the practice, and identify barriers affecting services.

**Materials and methods:**

Seven scientific electronic databases were searched. The review was conducted in accordance with PRISMA guidelines. This review was subjected to descriptive analyses, meta-analyses of proportion, and narrative synthesis. The Mixed Methods Appraisal Tool version 2018 was used to assess the quality of studies.

**Results:**

Minimal physician participation, community pharmacies' owners and managers, and gender imbalance among pharmacists all arose as gaps in Saudi Arabia. The findings showed that community pharmacies enable self-medication, with one-third of antibiotics being dispensed without prescriptions despite the threat of law enforcement. The deficiency of patient counseling by community pharmacists threatens patient safety, and the lack of knowledge about clinical services diminishes opportunities for adequate practice.

**Conclusion:**

Several factors contributing to this inadequate performance and low satisfaction levels were reported. The findings were alarming and highlighted the need to intensify efforts towards Saudi Vision 2030, which aims to improve the overall population health and healthcare system in Saudi Arabia. Our review suggests that more efforts are needed to integrate key commercial, administrative, and technical sectors associated with the processes of community pharmacy dispensing and counseling.

## 1 Introduction

Globally, community pharmacists are the third-largest group of healthcare professionals, behind only physicians and nurses.^1^ Their role extends far beyond dispensing medication: due to a rapid evolution of the healthcare system, community pharmacists are involved in a diverse range of advanced health service delivery. This evolution can potentially improve pharmaceutical care delivery, offering a positive outlook for the profession's future.^2^ The emerging community pharmacy (CP) model, conceived in industrialized countries, visualizes patient-centered service providers^3^ for services including public health screenings, medicine use reviews, and flu vaccination. These advanced services require a high level of clinical, research, and educational competence in community pharmacists.^4^

However, the services offered by CPs in developing countries are generally limited and tend to be associated with unique and often challenging sets of circumstances.^5^ The traditional view of CPs as intermediaries between physicians and patients means that patient-centered and advanced professional services are not fully implemented alongside traditional, product-centered services.^6^ Nevertheless, public health officials share an understanding of the need to expand the role of CPs in healthcare delivery. Such an expansion could potentially improve medication adherence, reduce hospitalizations, and bolster primary care health services, particularly in managing chronic diseases.^7^

In Saudi Arabia (SA), 23,649 community pharmacists work in 10,347 independent and chain CPs.^8^ Of them, 18,967 are expatriates, and 4682 are Saudi citizens.^8^ Regarding the profession, CPs are mainly regulated by three official authorities. The Saudi Ministry Of Health (MOH) is responsible for issuing and updating the national drug formulary, granting licenses to establish a CP premise, and releasing legislation and policies.^9^ The Saudi Food and Drug Authority is responsible for the safety of medicines and electronic health devices, including registration and price.^10^ The Saudi Commission of Health Specialties handles the registration and classification of healthcare-related practices, including pharmacists, enabling them to practice the profession.^11^

However, implementing advanced patient-centered CP services, including relevant legal aspects, is underway.^12^, ^13^, ^14^ At the same time, the CP sector is dominated by expatriate community pharmacists,^8^ which may lead to language and cultural miscommunications, affecting CPs' practice in the highly diverse Saudi population. Since its inception in 2016, Saudi Vision 2030 and the National Transformation Program have been instrumental in shaping the country's future. The vision is based on economic, social, and cultural pillars. The National Transformation program includes numerous initiatives aimed at transforming the current model of CP services^15^ by expanding community pharmacists' role to include patient-centered healthcare provision.^16^ Moreover, this program also contains a proposal to transfer all outpatient pharmacy services from the public sector to CPs.^16^ Therefore, in 2018, one key initiative is to start digitizing data and launch an electronic prescription platform (Wasfaty) so patients can receive their medication for free from optional CPs. This advanced digital, dynamic, and regulated platform ensures reduced or averted medication errors, drug-drug interactions, and duplicate dispensing. It also helps direct communication between community pharmacists and physicians.^17^

To this end, understanding the multiple stakeholders' views is essential to the health system transformation: relevant stakeholders from multiple sectors can contribute to the evidence base for Saudi CP services and the know-how of good pharmacy practices in line with Saudi Vision 2030. The current systematic review and meta-analyses aimed to identify all empirical research studies published between 2010 and 2020 in SA on CP services, which took the perspectives of multiple stakeholders, including CP users, community pharmacists, physicians, academics, and policymakers. This review specifically evaluated the services provided by community pharmacies and the satisfaction levels with those services, and it investigated any perceived barriers affecting their practice.

## 2 Materials and methods

### 2.1 Protocol and registration

This systematic review was guided by the preferred reporting items for systematic reviews and meta-analyses (PRISMA, 2020) guidelines, found in supplementary material 1. A review protocol for this review was published on the international Prospective Register of Systematic Reviews (PROSPERO), registration number #CRD42020202653, on September 4, 2020.

### 2.2 Search terms and strategy

The Setting, Population, Intervention, Comparison, and Evaluation (SPICE) conceptual framework was proposed to determine the search terms for this review.^18^ (see Table 1, Supplementary Material 2). This systematic review had eight categories of inclusion and exclusion criteria: type of published articles, participants, setting, location, study design, the language of published articles, publication date, and type of access to databases. Further details regarding the inclusion and exclusion criteria can be found in Table 2 of Supplementary Material 2. Search terms were identified by all reviewers using medical subject headings (MeSH) in PubMed and thereafter applied to all other compatible database searches. Seven scientific electronic databases were searched: CINAHL, Cochrane Library, Ovid, Google Scholar, PubMed, Scopus, and Web of Science. Each database search was subjected to specific search options and restrictions based on inclusion criteria (Table 3, Supplementary Material 2), and Boolean search techniques combined keywords by the operators ‘OR’ and ‘AND’. A pilot search was applied to evaluate each database's initial identification of relevant studies. An alert notification was established in all databases for any newly published studies which matched the inclusion criteria. The search of all combined terms and keywords is provided in Table 4, Supplementary Material 2.

### 2.3 Selection processes

The selection process began by identifying studies, followed by a screening process. The identification process was conducted in two phases: in Phase I, studies which met all inclusion criteria were identified, saved, and exported into the EndNote reference manager,^19^ and duplicate records were removed; in Phase II, studies were identified through citation searching of the included studies in Phase I. Studies identified in both phases were subjected to the screening process, which was conducted in two phases. In Phase I, the titles and abstracts of the identified studies were screened, and irrelevant studies were excluded. Each study's references were screened in order to identify studies through citation searching, and any title which included “Saudi” as a keyword was subjected to the screening process. In Phase II, the full texts of the remaining studies were assessed for eligibility, and records which did not match the inclusion criteria were excluded. The principal author (KA) conducted the selection process, and a second review author (MH) cross-checked the selection process following the same eligibility criteria; any disagreement was resolved by discussion or, where necessary, with the help of a third author (LH).

**Table 1:** Characteristics of included studies (*n* = 89).

Characteristics of included studies	Frequency	%
**Type of stakeholders**		
Community pharmacists^26^, ^27^, ^28^, ^29^, ^30^, ^31^, ^32^, ^33^, 34., 35, 36, 37, 38, 39, 40, 41, 42, 43, 44, 45, 46, 47, 48, 49, 50, 51, 52, 53, 54, 55, 56, 57, 58, 59, 60, 61, 62, 63, 64, 65, 66, 67, 68, 69, 70, 71, 72, 73, 74, 75, 76, 77, 78, 79, 80, 81, 82., 83, 84, 85, 86, 87, 88, 89, 90, 91, 92, 93	68	76.40 %
Patients or customers^94^, ^95^, ^96^, ^97^, ^98^, ^99^, ^100^, ^101^, ^102^, ^103^, ^104^, ^105^, ^106^, ^107^, ^108^, ^109^	16	17.80 %
Multiple stakeholders^12^^,^^110^, ^111^, ^112^, ^113^	5	5.60 %
**Type of research design**		
Quantitative approach	75	84.30 %
Cross-sectional surveys	59	66.30 %
Simulated patient visits	17	19.10 %
Qualitative approach (interviews)	12	13.50 %
Mixed-method approach	2	2.20 %
**Provincial distribution**		
Riyadh	36	40.45 %
Eastern province	14	15.73 %
Makkah	11	12.35 %
Asir	5	5.61 %
Al Madinah	4	4.50 %
Al Qassim	4	4.50 %
Hail	1	1.12 %
Multiple province or cities	8	8.98 %
Nationwide	6	6.74 %
**Details of dimensions**		
OTC dispensation and self-medication	18	20.22 %
Dispensation of generics, prescription-only medicines, and antibiotics	18	20.22 %
Dispensing and counseling practices for specific cases	7	7.87 %
Counseling for various health conditions	12	13.48 %
Patient safety measures	18	20.22 %
Clinical services	11	12.36 %
Satisfaction levels	5	5.62 %
Community pharmacists' satisfaction	2	2.24 %
Customers' satisfaction	3	3.37 %
**Quality assessment**		
Low (50 %)	2	2 %
Moderate-low quality (51*–*65 %)	25	28 %
Moderate-high (66*–*79 %)	38	43 %
High (≥ 80 %)	24	27 %

**Table 2:** The reasons for dispensing POMs and antibiotics without prescription by community pharmacists.

Reasons	N	Events	Total	Proportion	95 % CI
If not dispensed, the patient can obtain it from any CP	3	255	307	0.869	[0.578, 1000]
Patient’ socioeconomic status	3	330	583	0.719	[0.246, 0.998]
Simple symptoms	3	254	652	0.678	[0.112, 1000]
Pharmacists' confidence/ knowledge	5	378	832	0.648	[0.339, 0.902]
Difficulties in reaching the clinic	3	291	654	0.631	[0.242, 0.941]

N = Total number of included studies.

**Table 3:** The barriers to patient counseling by community pharmacists.

Barriers	N	Events	Total	Proportion	95 % CI
Lack of time	8	1511	2990	0.494	[0.346, 0.643]
Lack of reliable sources /information	4	638	2481	0.222	[0.102, 0.371]
Patient culture/not interested	5	171	861	0.202	[0.047, 0.424]
Lack of pharmacists' confidence/ knowledge	7	491	2895	0.179	[0.075, 0.313]
Community pharmacists are not interested	5	213	2267	0.102	[0.035, 0.194]

N = Total number of included studies.

**Table 4:** The barriers to ADRs reporting system by community pharmacists.

Barriers	N	Events	Total	Proportion	95 % CI
Reporting forms are not available	4	219	374	0.678	[0.359, 0.926]
No motivation	3	72	204	0.387	[0.175, 0.624]
Reporting is time consuming	4	109	374	0.307	[0.233, 0.386]
Fear of legal liability	3	59	204	0.287	[0.082, 0.551]
I am not sure if it's ADRs	3	62	204	0.280	[0.000, 0.756]
Forms are too complicated	4	89	374	0.236	[0.194, 0.282]
Insufficient clinical knowledge	4	78	374	0.189	[0.058, 0.370]

N = Total number of included studies.

### 2.4 Data collection process

A data collection form, piloted with approximately 10 % of the studies, was created to extract the following items from each study: reference, location, aim, study design, sample size, key findings, and mixed methods appraisal tool (MMAT) score. Individual studies' characteristics are listed separately, based on participant types and outcomes, in chronological order. The data were extracted by KA and cross-checked by MH following the same eligibility criteria process, and differences were resolved by reaching a consensus (Supplementary Material 3).

### 2.5 Quality assessment

The MMAT version 2018 assessed the quality of all included studies.^20^ The MMAT contains three sets of criteria, one per type of research method (qualitative, quantitative, or mixed methods). Each study was judged on seven methodological criteria; according to the MMAT, a ‘Yes’ response refers to a criterion's presence, a ‘NO’ response refers to its absence, and a ‘Cannot tell’ response indicates that the paper does not report sufficient information to answer or that it reports unclear information. For each criterion, ‘Yes’ and ‘No’ responses were scored 1 point and 0 point, respectively, while a ‘Cannot tell’ response was scored 0.5 points. Each study's score was calculated using the equation [Total points earned ÷ 7 (Total points possible) × 100 = Scoring %]. The quality score of each study was classified as low (≤ 50 %), moderate-low (51*–*65 %), moderate-high (66*–*79 %), or high (≥ 80 %). Two independent reviewers (KA and MH) conducted the quality assessment of all included studies, and the results were then discussed with (LH) to ensure consensus. A standardized Microsoft Excel spreadsheet was created for the quality assessment (Supplementary Material 4).

Studies with low or moderate-low quality (ranging from 50 % to 65 %) were not excluded from the current analyses. This decision ensured that no potentially valuable data would be lost, as even lower-quality studies can provide meaningful information.

### 2.6 Data management, statistical analyses, and synthesis methods

Graphical and numerical descriptive statistics were used to summarize the frequencies and percentages of study characteristics, quality assessment, and key outcomes of CP services. In addition, an analytical statistical assessment was performed using a meta-analysis of proportions utilizing JBI SUMARI software.^21^^,^^22^ Freeman-Tukey transformation and a random effect model were applied, and the 95 % confidence interval (CI) was estimated. Since different studies utilized different measurement scales to report barriers and reasons for the practice, a cut-off of three or more eligible quantitative studies reporting a similar measurable outcome were included in the analyses. For example, we considered statements such as agreement scores (strongly agree, agree) as “Yes” and disagreement scores (strongly disagree, disagree) as “No”; similarly, ‘always’, ‘often’, ‘sometimes’, and ‘rarely’ were assigned “Yes”, while ‘never’ was assigned “No”. Regarding CP services and practice, the eligible quantitative studies reporting a similar measurable outcome in observational studies and simulated patient visits (SPV) were used for meta-analyses. The SPV is a method involving a trained individual who convincingly simulates a standardized scripted request as a realistic patient^23^; the analysis of these studies considers their direct observations of events and behaviors performed in non-controlled settings to identify gaps in evidence-based practice implementation.^24^^,^^25^ A narrative synthesis approach was used to report and discuss the main findings.

## 3 Results

**Fig. 1:**
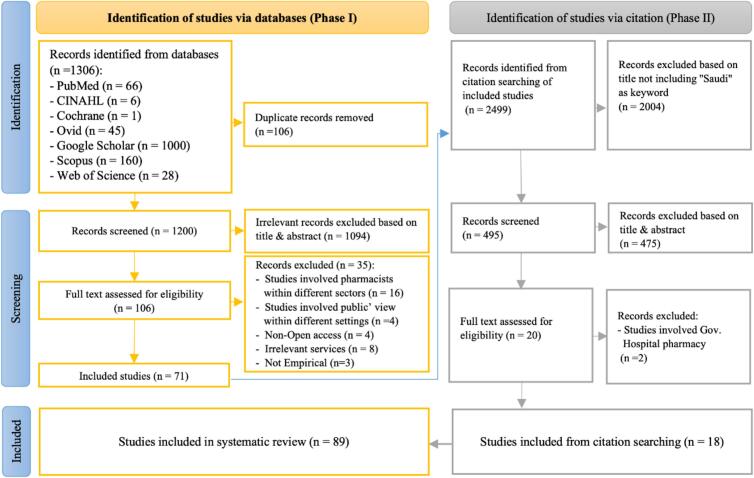
PRISMA flow diagram for systematic review.

The study selection process initially yielded 3805 studies through the two main phases. In Phase I, 1306 records were identified from the databases, with 71 studies included; in Phase II, 2499 records were identified from citation searching of 71 studies in Phase I, with 18 additional articles. Thus, 89 studies from the two phases were included in the systematic review. Fig. 1.

### 3.1 Characteristics of included studies

Five key dimensions of CP services and practices were derived from the 89 included studies. These dimensions are medication dispensing, patient counseling, patient safety measures, clinical services and satisfaction level. Table 1.

### 3.2 Medication dispensing

#### 3.2.1 Over the counters (OTCs)

A survey study in Riyadh city in 2012 revealed that 70 % of community pharmacists knew the OTC directory,^114^ but a more recent study in Riyadh in 2020 suggested that only 40 % of them dispensed OTCs according to evidence-based practice.^67^ Herbal medicine dispensation was a common practice of community pharmacists, with poor awareness regarding potential herb-drug interactions.^59^^,^^61^^,^^63^^,^^64^ Two studies explored dispensing vitamin and nutritional supplements and found this to be a common practice, with adequate scientific knowledge.^65^^,^^69^ There is a lack of basic knowledge of weight-reduction (diet) products among pharmacists, who believe these products are effective and safe, as one study reported.^62^ Proton pump inhibitors (anti-ulcer medicines or stomach acidity reducers) were addressed in only one study, and most pharmacists claimed that they knew about their indications and side effects, with 94 % of them dispensing proton pump inhibitors to their patients.^66^ Oral healthcare was examined by two studies, which found a lack of oral health knowledge among pharmacists.^72^^,^^115^ One study evaluated the awareness level of sunscreens and moisturizer products, revealing that most pharmacists need more understanding of cosmetics.^68^

#### 3.2.2 Self-medication

Self-medication patterns were discussed in six studies. One study, exploring the topic from pharmacists' perspectives, showed that self-medication is more common among men than women,^116^ with similar findings across four studies examining the public perspective.^45^^,^^100^, ^101^, ^102^ People with low education levels and non-healthcare professionals were more likely to prefer self-medication.^45^^,^^101^ The overall proportion of self-medication for minor symptoms by the public was 0.712 with 95 % CI [0.495, *0.886*] presented in Table 5. More details in Fig. 1, Supplementary Material 5.

#### 3.2.3 Generic medicines

Most pharmacists prefer to dispense generic medicines (i.e., drugs with the same active-ingredient formula as brand-name drugs), but they need to know more about the efficacy and safety profiles of such medicines. According to retrieved studies, cutting costs for the customer based on physical appearance and product bonuses offered by pharmaceutical companies were the main factors behind community pharmacists dispensing generic medicines.^37^, ^38^, ^39^

**Table 5:** The overall meta-analyses of proportion of Saudi CP practice 2010–2020.

Practice	N	Events	Total	Proportion	95 % CI
Public practiced self-medication for minor symptoms	5	2678	3723	0.712	[0.495–0.886]
Pharmacists dispensed POMs without prescription	4	491	863	0.717	[0.497–0.894]
Pharmacists dispensed antibiotics without prescription (before it was outlawed)	4	447	541	0.931	[0.795–0.999]
Pharmacists dispensed antibiotics without prescription (after it was outlawed)	3	142	548	0.336	[0.001–0.857]
Pharmacists asked about associated symptoms	4	787	1468	0.557	[0.408–0.701]
Pharmacists asked about concomitant drugs/comorbidities	4	307	1264	0.151	[0.037–0.320]
Pharmacists asked if the patient had taken this medicine before	4	72	769	0.096	[0.049–0.156]
Pharmacists asked about drug allergy	6	72	1696	0.046	[0.013–0.097]
Pharmacists asked about pregnancy status	3	29	279	0.251	[0.001–0.664]
Pharmacists provided duration of use	4	300	1090	0.276	[0.026–0.654]
Pharmacists demonstrated the correct use of metered dose inhaler steps	3	18	335	0.049	[0.021–0.086]
Pharmacists' familiarity with the ADRs reporting system	6	231	577	0.384	[0.135–0.670]

N = Total number of included studies.

#### 3.2.4 Prescription only medicines (POMs)

Malpractice in drug dispensation was observed in 15 studies. POMs, including antibiotics, are medicines which cannot otherwise be dispensed without an authorized prescription.^40^, ^41^, ^42^^,^^44^, ^45^, ^46^^,^^48^^,^^49^^,^^51^^,^^54^^,^^94^^,^^95^^,^^117^, ^118^, ^119^ Three studies evaluated the impact of law enforcement found that antibiotics continue to be dispensed without a prescription by community pharmacists.^56^, ^57^, ^58^ The overall proportion of dispensing POMs was 0.717 with 95 % CI [0.497, 0.894]; the proportion of dispensing antibiotics without a prescription before it was outlawed was 0.931 with 95 % CI [0.795, 0.999], and after it was outlawed, the proportion was 0.336 with 95 % CI [0.001, 0.857]. More details are in Figs. 2 and 3, Supplementary Material 5. Five studies listed the reasons reported most frequently by community pharmacists for dispensing POMs, including antibiotics.^41^^,^^42^^,^^46^^,^^51^^,^^118^ Table 2.

### 3.3 Patient counseling

Several retrieved studies evaluated the quality of community pharmacists' patient counseling for multiple health conditions.^26^, ^27^, ^28^, ^29^, ^30^, ^31^^,^^47^^,^^92^ Their findings revealed poor recognition among community pharmacists of the use of Metered Dose Inhalers,^34^^,^^35^^,^^93^^,^^120^ while one study showed community pharmacists were competent enough in counseling asthma patients.^34^ Interestingly, out of the 89 included studies, only one interventional study found that clinical programs by community pharmacists to counsel people with diabetes were effective in raising patients' awareness.^113^

One study concluded that the most relevant counseling attributes from the public's perspective were the quality of the information provided during counseling, the pharmacist's experience and education, and the explanation of associated side effects and drug-drug interactions.^96^ According to the meta-analyses, the highest proportion of questions by community pharmacists during patient counseling was for associated symptoms (0.557), and the lowest was for asking about drug allergies (0.046). More details are in Figs. 4 and 5, Supplementary Material 5. Eight studies reported the top five barriers to patient counseling for various health conditions among community pharmacists.^26^^,^^27^^,^^36^^,^^64^^,^^72^^,^^78^^,^^121^^,^^122^ The barriers to patient counseling are presented in Table 3.

### 3.4 Patient safety measures

Two studies investigated the proportions of errors in dispensing medications by community pharmacists. The top five factors contributing to dispensing errors were look-alike and sound-alike medicines (e.g., visually similar packaging or similar phonetics), fatigue, generic medicines, lack of privacy when dispensing, and interruptions.^73^ Prescribing errors in physicians' prescriptions were reported to be common. The quality of written prescriptions was deficient in some elements (e.g., patient name and age, date, and diagnosis).^75^

One study shed light on the ethical issues for community pharmacists, including dispensing doses outside the national formulary and controlled drugs, resulting in a need for more knowledge on these ethical issues.^122^ According to another study, 36 % of community pharmacists believe administering controlled medications without a prescription is necessary for their income, and 14 % reported a complete absence of monitoring of their services by authorities.^77^ The antimicrobial stewardship program in CP was found to be substandard, indicating a need for significant improvement in this area.^55^ Two other studies focused on CPs' preventive measures during the COVID-19 pandemic. One study, which used self-administered surveys, demonstrated readiness among pharmacists^82^; by contrast, the other study, which used a SPV, shown poor compliance with preventive measures against COVID-19, underscoring the need for better adherence to safety protocols.^83^

One study evaluated medication safety during pregnancy; in this respect, community pharmacists did not always offer correct advice to pregnant women or ask female patients about their pregnancy status.^74^ Another study evaluated common potential drug-drug interactions among 26 drug pairs and found that community pharmacists' knowledge on this topic needed to be improved.^76^

Two studies explored medication safety problems; factors contributing to medication safety problems included commercial pressures on community pharmacists by pharmaceutical companies, a lack of enforcement of regulations, the fragmented healthcare system (control, regulation, and lack of patient database), and self-medication.^111^ In addition, the priorities of medication safety problems were lack of pharmacy counseling area, lack of communication with physicians, lack of patient databases, lack of post-registration pharmacist education, and long working hours.^110^

Only half of community pharmacists screened for the associated risk factors of nonsteroidal anti-inflammatory drugs for their patients and provided advice regarding adverse drug reactions (ADRs).^32^ Seven studies on the community pharmacists' perspective revealed a poor understanding of the ADR reporting system.^78^^,^^86^, ^87^, ^88^, ^89^, ^90^ A nationwide study involving 5228 participants on the public's perspective estimated the prevalence and awareness of ADR reporting^97^: of the participants, 28 % experienced an ADR over the course of a year; of those who experienced an ADR, only 30.26 % were aware of the reporting system, and only 14.29 % filed a report. According to this review, the overall proportion with respect to community pharmacists' familiarity with the reporting system was 0.384 with 95 % CI [0.135, 0.670]. More details are in Figs. 6 and 7, Supplementary Material 5. Four studies reported the barriers to ADR reporting which were shared by community pharmacists.^86^^,^^87^^,^^89^^,^^90^ Table 4.

### 3.5 Clinical services

As determined from the retrieved studies, many critical clinical services were lacking and did not meet international standards, with no computerized documentation system in place.^78^ Additionally, the current practice of Saudi CPs is product-oriented, rather than focused on actual pharmaceutical care.^12^ Approximately 53 % of community pharmacists thought they were knowledgeable about the concept of pharmaceutical care.^79^ As a result, effective clinical services are bound by community pharmacists' knowledge, needs, values, and trust; therefore, 66 % of them denied practicing clinical services.^80^ In terms of the public's perspective, chronic disease patients reported not having experienced such clinical services and a lack of trust in community pharmacists^104^^,^^123^; additionally, 96 % of the Saudi public perceived medication-therapy management service as beneficial for patients' care.^109^ One study evaluated the association between diabetic patients' knowledge and their medication adherence and found that half of those with disease knowledge were more likely to have high adherence.^98^

Two studies reported on the provision of immunization services in CPs. One found that 45 % of community pharmacists were unwilling to provide immunization services for various reasons, including lack of training, concerns about patient safety, and the resultant increased workload.^81^ For their part, the public expressed a need for immunization services, but there were concerns about pharmacists' training, the lack of a private area to conduct the service, the gender of the pharmacist, and the cost of the service.^105^ Finally, only one study highlighted the importance of interprofessional collaboration between physicians and community pharmacists regarding clinical services: while community pharmacists would like to have better relationships with physicians, physicians need to be made aware of the role of community pharmacists.^112^

### 3.6 Satisfaction level

Two studies measured job satisfaction among community pharmacists. Pharmacists who worked in independent CPs had lower satisfaction levels^84^; long working hours, workload, duty shifts, difficulty obtaining personal or sick leave, and low salaries were job stressors related to patient care responsibility.^84^^,^^85^ The professional performance of community pharmacists was below expectations according to three studies involving the public: 37 % perceived community pharmacists as mere vendors, and 48 % did not receive enough counseling.^106^^,^^107^ Only 41 % of the public reported being satisfied with overall services,^107^ and only 35.3 % of Saudi women were satisfied with male community pharmacists' services, while 63 % found it embarrassing to discuss issues with male community pharmacists.^108^ Table 5 illustrates the overall meta-analyses of the proportion of Saudi CP practice 2010–2020.

## 4 Discussion

This systematic review is timely and crucial for the Saudi transformation program based on Saudi Vision 2030, which expands the role of CPs to include patient-centered healthcare provision: the national transformation plan contains a proposal to transfer all outpatient pharmacy services from the public sector to CPs.^16^ The review's key findings revealed the prevalence of the practice of self-medication, malpractice in dispensing POMs, a deficiency in patient counseling, and many factors contributing to patient safety problems and low satisfaction levels due to the lack of patient-centered care.

The results of 89 research studies reveal an urgent need for more trial studies and interventional studies, as well as a lack of the perspectives of physicians, female community pharmacists, and CP owners and managers. However, a noticeable trend was observed in conjunction with the launch of Saudi Vision 2030 in 2016, whereafter both methodological quality and publishing quantity of studies increased notably, involving more public and other stakeholders. Regardless, most community pharmacists were male. The possible reason for the absence of women's participation is that pharmacy colleges for women in SA were only opened recently, leading to a persistent gender imbalance in the CP workforce. In addition, female pharmacists prefer working in the government sector, for personal and household stability reasons.^14^ Most Saudi women were unsatisfied with CP services and found it embarrassing to discuss issues with male community pharmacists, necessitating the empowerment of female pharmacists in CP in SA.^13^^,^^14^^,^^108^

Self-medication is a widespread global phenomenon in which individuals take non-prescription medication without the advice of a healthcare professional or prescription, which may lead to health problems such as ADRs or drug-drug interactions.^124^ The review's findings show that people in SA often practice self-medication for minor health issues; however, the retrieved studies suggest that stakeholders should address the factors which lead to self-medication—including avoiding long waits at clinics and hospitals, seeking quicker relief, and the potential cost savings—revealed in several studies.^70^^,^^100^, ^101^, ^102^ Additionally, the need for local health authorities to raise public awareness about the danger of self-medication may enhance patient health literacy and limit this phenomenon. The OTC directory and pharmacist prescriptions should be re-evaluated, due to conflicts with available guidelines.^67^ Implementing evidence-based practice guidelines for OTC medications can support community pharmacists' practice and improve health outcomes.^67^

In 2018, the Saudi MOH released an official law to reduce the widespread practice of dispensing POMs, including antibiotics. This malpractice is credited with the rise of certain global public health problems, such as microbial resistance, placing a financial burden on healthcare systems and patients. In addition, OTC dispensing of antibiotics can lead to inappropriate use, such as for viral infections (e.g., colds and flu), where they are ineffective, potentially contributing to side effects.^124^ According to the review's findings, community pharmacists claimed that patients would easily obtain antibiotics from other CP sources despite being prohibited by some CPs. Another main reason was a patient's economic status—i.e., some patients cannot afford to visit a physician. However, these reasons were reported in several studies before the law came into force. Nevertheless, some community pharmacists still violate the law, with one-third of antibiotics dispensed without a prescription. Conceptually, community pharmacists' practices with respect to antimicrobial stewardship application in SA are poor.^55^ Addressing possible cultural and social barriers requires a multifaceted approach, including public awareness campaigns, education on the appropriate use of antibiotics, and consolidated monitoring of antibiotic sales, which would likely limit this phenomenon.^48^^,^^56^

Patient counseling, the backbone of pharmaceutical care, involves providing information, advice, and support to assist patients with their medication use. Evidence indicates that counseling by community pharmacists improves clinical outcomes.^27^ However, this review predominantly revealed a deficiency in patient counseling. At the local level, most of the public receives suboptimal counseling at the cashier counter and in the presence of other nearby customers.^90^^,^^106^^,^^107^^,^^109^^,^^125^ Counseling deficiencies among community pharmacists could seriously impact their ability to provide adequate overall pharmaceutical care, and insufficient continuing professional development, reliable sources, and support from CP management in the face of high workload and stress lead to low health outcomes.^126^, ^127^, ^128^ Ensuring that community pharmacists are well-equipped with counseling skills and therapeutic knowledge requires the classification and registration of community pharmacists by the Saudi Commission for Health Specialties to be reviewed and re-evaluated. More importantly, policies and guidelines for counseling practice need to be implemented, so as to standardize the level of services provided by CP.^31^^,^^126^ Additionally, the barriers to patient counseling reported in this review—the main factors being lack of time and reliable sources—must be addressed by CP owners and managers.

Medication errors can occur at any stage of the medication delivery process, and they compromise patient safety. According to a recent international systematic review, contributing factors to such errors include inadequate training, knowledge gaps, and high workloads for healthcare practitioners.^129^ Similar to global findings, the common factors leading to medication errors in Saudi CPs were look-alike/sound-alike medicines, generic medicines, lack of privacy when dispensing, and interruption while counseling.^73^^,^^110^ Additionally, prescribing errors are common in physicians' prescriptions and are associated with low-quality, handwritten prescriptions which lack essential information such as the patient's name, age, date, and diagnosis.^75^ To this end, computerized medication prescriptions—such as the national electronic prescription ‘Wasfaty’, supported with software to detect drug-drug interaction—would be vital to reducing medication errors.^14^ The monitoring of medication safety standards by local authorities would ensure that community pharmacists are continually trained to prescribe and dispense appropriate medicines safely.

Medication errors exist in relationship to ADRs.^130^ The meta-analysis results indicate that most community pharmacists needed a better understanding of the ADR reporting system process. This poor understanding also extends to the public, who needed to be made aware of the reporting system.^97^ However, most community pharmacists claimed that the reporting form was unavailable, the forms were too complicated, or they were afraid of legal liability.^86^^,^^87^^,^^89^^,^^90^ Although the National Pharmacovigilance and Drug Safety Centre offers online guidance on ADRs and events, educational programs and awareness campaigns should continue to advocate for ADR reporting by community pharmacists and the public.^131^

Steps have been taken in many industrialized countries to equip community pharmacists with key responsibilities and provide integrated healthcare services, aligning with the United Nations Sustainable Goals 2030. For example, community pharmacists in Australia, the United Kingdom, and the United States manage chronic diseases. Additionally, pharmacists can provide public health programs such as smoking cessation and bodyweight management as part of their essential practice. Public health screenings (e.g., vital signs, blood glucose and blood pressure measurements) can also be provided by CPs.^132^ One key issue which needs to be addressed in the National Transformation Program is the need for more patient-centered care in Saudi CPs. Many of these essential clinical services need to be improved or introduced.^78^ However, only half of community pharmacists knew about clinical services, leading to missed opportunities to practice these services.^80^ However, the Saudi MOH recently introduced a package of CP services.^133^ A pharmacist with a certificate in pharmacy-based immunization delivery can deliver several vaccinations. In addition, some public health screening services are possible, including measuring blood pressure, heart rate, body temperature, and body mass index.^134^ Clinical pharmacists can also treat some minor illnesses and offer medication therapy management.^135^

However, factors such as long working hours, managerial duties, limited personal leave, and low salaries may hinder community pharmacists from providing clinical services, as this review indicated.^85^^,^^136^ According to recent local studies, more factors may affect CP performance and quality of service; concluded that 20 factors, falling into four main dimensions (personal, contextual, operational, and healthcare system), might affect CPs' ability to deliver such services, with their top factors being inadequate pharmaceutical care training, lack of collaboration with physicians, lack of a pharmacy clinic, and lack of access to patients' medical records.^14^^,^^137^ In any case, members of the public with chronic diseases reported not having experienced clinical services^104^^,^^123^; consequently, the professional performance of community pharmacists was below expectations in the public's view.^106^^,^^107^

Some possible strategies to overcome these barriers to offering specialized services are enhancing community pharmacists' clinical knowledge and skills through specific training in clinical skills, patient education programs on the rational use of medicines, improvements in interdisciplinary collaboration with physicians via a clear policy, and effective cooperation between health authorities to successfully transform practice.^12^^,^^78^^,^^80^^,^^112^ At this point, a multi-stakeholder approach for developing a national system of measurable quality indicators for CP care is urgently needed.^138^ Recently, the International Pharmaceutical Federation has developed the Global Competency Framework for effective and sustained performance and advanced practice.^139^ Therefore, implementing standards for accreditation could create a competitive environment for CP, granting them service remuneration and enhancing customers' confidence in the quality and safety of pharmaceutical care.^137^

## 5 Strengths and limitations

This is the first systematic review and meta-analyses to involve a multi**-**stakeholder perspective of quantitative and qualitative approaches towards CP services in SA. Using the mixed methods approach provides a better understanding of research problems and complex phenomena.^140^ It puts findings into context, and allows to explore different dimensions in the transition of CP services towards a more patient-centered model. Nevertheless, the period of this review should be considered as a baseline for performance assessment that can be used to assess change over time. This review summarized the level of Saudi CP services and the extent of satisfaction with services, and it investigated barriers to adequate practice, from 2010 to 2020; the goal of this was to empower decision-makers, CP owners, CP managers, and community pharmacists to take the necessary steps to improve service performance.

In this review, we have included several studies of low and moderate-low quality. However, including all eligible studies, regardless of quality, can provide a more comprehensive overview of the existing evidence on topic,^141^ as including all studies and then transparently assessing their quality can provide a balanced view.^142^ High heterogeneity is expected in meta-analyses, due to differences in studies' populations, methodologies, settings, and time periods.^141^ A random-effects model was used to account for this heterogeneity, since no subgroup analysis was possible, and meta-regression was not considered due to small studies size.^143^ Publication bias, funnel plots, sensitivity, and specificity tests are neither common nor recommended for proportional meta-analyses and might not be suitable or informative for proportional data^144^; therefore, we did not conduct any of these tests.

## 6 Conclusion

The services provided by Saudi CPs in 2010–2020 were below expectations. Self-medication for minor ailments was common among the public, and CPs dispensed antibiotic drugs without a prescription. Medication safety problems associated with patient counseling and medication delivery issues were observed. A lack of knowledge about clinical skills leads to a lack of practice in clinical services. Low job satisfaction among community pharmacists could hinder services. Given the inadequate performance of CP services—which fall outside the national transformation plan for CP partnerships providing outpatient pharmacy services—the findings of this review sound the alarm about the urgency of intensifying efforts to align with Saudi Vision 2030. More studies are needed for further assessment.

The following are the supplementary data related to this article.

Supplementary material 1 PRISMA 2020 checklist. Supplementary material 1

Supplementary material 2 Systematic search strategy. Supplementary material 2

Supplementary material 3 Summary of studies' characteristics and key findings. Supplementary material 3

Supplementary material 4 Quality assessment. Supplementary material 4

Supplementary material 5 Forest plots of meta-analysis. Supplementary material 5

## CRediT authorship contribution statement

**Khalid S. Alghamdi:** Writing – review & editing, Writing – original draft, Visualization, Validation, Methodology, Investigation, Formal analysis, Data curation, Conceptualization. **Max Petzold:** Writing – review & editing, Supervision, Methodology, Conceptualization. **Mahdi H. Alsugoor:** Writing – review & editing, Validation, Methodology, Formal analysis, Data curation. **Hafiz A. Makeen:** Writing – review & editing, Validation, Methodology. **Christina Ljungberg Persson:** Writing – original draft, Supervision, Methodology, Formal analysis. **Laith Hussain-Alkhateeb:** Writing – review & editing, Validation, Supervision, Methodology, Investigation, Formal analysis, Data curation, Conceptualization.

## Funding

This systematic review is a part of PhD project sponsored by Al Baha University, Saudi Arabia.

## Declaration of competing interest

The authors declare that they have no known competing financial interests or personal relationships that could have appeared to influence the work reported in this paper.
