# Supplementary material for: Multi-stakeholder perspective on community pharmacy services in Saudi Arabia: A systematic review and meta-analyses for 2010–2020
Source: Explor Res Clin Soc Pharm. 2025 Apr 28;18:100608. doi: 10.1016/j.rcsop.2025.100608 (PMC12099458; doi:10.1016/j.rcsop.2025.100608)
Supplement: Supplementary material 2 — Systematic search strategy. [file mmc2.pdf]

Supplemental material 2. Systematic search strategy.

Table 1. Main concepts using SPICE framework. *(C, Comparison is not applicable)*:

**Setting**	**Population**	**Intervention**	**Evaluation**
Community Pharmacy	**Multi-stakeholders:**	Community Pharmacy Services.	Perspective: Expectation, Experience, Feedback
Saudi Arabia	Public/Patients/ Users/ Customers	Pharmacy Practice/ Pharmaceutical Care	Knowledge, Attitude, Practice/Perception,
	Community Pharmacists	Healthcare/ Public Health	Opinion, Views, Behaviour, Belief, Awareness, Satisfaction
	Health professionals/ Policymakers		Barriers, Facilitators.

Table 2. Inclusion and exclusion criteria:

**Category**	**Inclusion Criteria:**	**Exclusion Criteria:**
Publication	Empirical peer-reviewed journal articles.	Grey literature, Editorials, Abstracts, Posters, Dissertation, Thesis or Review.
Participants	- Patients who visited community pharmacies or customers/users<br>- Community pharmacists & staff members.<br>- Stakeholders' perspective on community pharmacy services.	- Pharmacists do not work in community pharmacies.<br>- Multi-Stakeholders' perspective on setting other than community pharmacy.
Setting	Conducted in community pharmacies including:<br>- Independent or chain community pharmacies.<br>- Affiliated or non-affiliated to (private) hospital/ Dispensary.	- Inpatient/outpatient hospital pharmacies in the governmental sector.<br>- Other pharmaceutical industries.
Location	Saudi Arabia.	Outside Saudi Arabia.
Study design	Empirical qualitative, quantitative, or mixed methods research studies.	Non-empirical research studies.
Language	English language only.	Studies wrote in any language other than English.
Date	2010-2020	Before 2010 and after 2020
Access	Open access with full-text articles	Non-open access or abstract only.

**Citation searching of included studies that are identified from databases are subjected to these criteria*

Table 3. Selected databases, search options and restrictions:

#	Database	Search options & Restrictions
**1**	**CINAHL**	Search options:<br>- Selected Database: CINAHL with full text<br>- Advanced Search<br>- AB Abstract<br>- Find all my search terms<br><br>Limit to:<br>- Peer-reviewed<br>- Research Article<br>- Published Date: January 2010 – December 2020<br>- English Language<br>- Human.<br>- Sex: All<br>- Age group: All<br>- Publication Type: Journal Article<br>- Special Interest: All<br>- English Language
**2**	**Cochrane Library**	Search options:<br>- Advanced Search, search manager<br>- Title, Abstract, Keywords<br>- Search word variations<br><br>Limit to:<br>- Content type: Trials<br>Publication year: January 2010 – December 2020
	**Ovid**	Search options:<br>- Resource selected: All Resources<br>- Multi-Field Search<br>- Keyword<br>- Map Term to Subject Heading<br><br>Limit to:<br>- Articles with Abstract<br>- English Language

3		- Humans<br>Publication Year: January 2010 – December 2020
4	**Google Scholar**	Search options:<br>- Advanced Search.<br>- With the exact phrase.<br>- Return articles dated between January 2010 – December 2020<br>- Sort by relevance.<br>- Page Search: 100 pages as a cut-off point.
5	**PubMed**	Search options:<br>- Advanced Search<br>- Field options: Title/Abstract<br><br>Limit to:<br>- Publication date: Custom Range 10 years: 2010/01/01- 2020/12/31<br>- Article Type: Journal article, Clinical Article, RCTs.<br>- Species: Human.<br>- Language: English.
6	**Scopus**	Search options:<br>- Advanced documents Search.<br>- Field options: Article title, Abstract, Keywords.<br><br>Limit to:<br>- All open access.<br>- Date range: 2010 -2020<br>- Added to Scopus in the last: 7 days<br>- Document type: Article<br>- Access type: All<br>- Keywords: Human, Articles<br>- Country: Saudi Arabia<br>- Source type: Journal<br>- Language: English

**7**	**Web of Science**	Search options:<br>- Selected database: All databases.<br>- Advanced Search<br>- Field Tags: TS=Topic (Title, Abstract, Author Keywords & Keywords Plus®)
		Limit to:<br>- Documents Type: Article.<br>- Language: English.<br>- Timespan, Custom year range: 2010-2020

Table 4. Search strategy using PubMed.

#	Keyword/s or Search term/s	Results
#1	("Community Pharmacy"[tiab] OR "Community Pharmacies"[tiab])	2,783
#2	("Retail pharmacy"[tiab] OR "Retail pharmacies"[tiab])	285
#3	("Pharmacy"[Mesh] OR "Pharmacies"[Mesh] OR "Pharmacy"[tiab] OR "Pharmacies"[tiab])	18,571
#4	#1 OR #2 OR #3	18,571
#5	("Saudi Arabia"[Mesh] OR "Saudi Arabia"[tiab] OR "Kingdom of Saudi Arabia"[tiab])	8,293
#6	"Public"[tiab]	186,559
#7	("Patients"[Mesh] OR "Patients"[tiab] OR "Patient"[tiab])	2,446,335
#8	("Customers"[tiab] OR "Customer"[tiab])	2,573
#9	("Consumer"[tiab] OR "Consumer"[tiab])	15,158
#10	("Clients"[tiab] OR "Client"[tiab])	18,794
#11	("Pharmacy Users"[tiab] OR "Users"[tiab])	59,246
#12	("Pharmacists"[Mesh] OR "Pharmacists"[tiab] OR "Pharmacist"[tiab])	14,040
#13	("Community Pharmacists"[tiab] OR "Community Pharmacist"[tiab])	1,537
#14	"Policymakers"[tiab]	8,875
#15	("Health Professionals" OR "Health Providers"[tiab])	25,270
#16	("Stakeholder Participation"[Mesh] OR "Stakeholders"[tiab])	21,276
#17	#6 OR #7 OR #8 OR #9 OR #10 OR #11 OR #12 OR #13 OR #14 OR #15 OR #16	2,667,728
#18	"Health Knowledge, Attitudes, Practice"[Mesh]	64,772
#19	"Knowledge"[tiab]	265,268
#20	("Attitude to Health"[Mesh] OR "Attitude of Health Personnel"[Mesh] OR "Attitude"[tiab] OR "Attitudes"[tiab])	272,954
#21	("Perception"[Mesh] OR "Perception"[tiab] OR "Perceptions"[tiab] OR "Perceived"[tiab])	292,277
#22	("Behavior"[Mesh] OR "Behavior"[tiab] "Behaviors"[tiab] OR "Behaviour"[tiab] OR "Behaviours"[tiab])	116,632
#23	("Awareness"[Mesh] OR "Awareness"[tiab] OR "Awarenesses"[tiab])	71,743
#24	("Public Opinion"[Mesh] OR "Opinion"[tiab] OR "Opinions"[tiab])	46,371
#25	("Perspective"[tiab] OR "Perspectives"[tiab])	127,225
#26	("View"[tiab] OR "Views"[tiab])	92,463
#27	("Patient Satisfaction"[Mesh] OR "Satisfaction"[tiab])	90,468
#28	("Expectation"[tiab] OR "Expectations"[tiab])	31,499
#29	("Experience"[tiab] OR "Experiences"[tiab])	299,554
#30	("Belief"[tiab] OR "Beliefs"[tiab])	35,900
#31	("Contribution"[tiab] OR "Collaboration"[tiab])	107,389
#32	"Feedback"[tiab]	46,909
#33	#18 OR #19 OR #20 OR #21 OR #22 OR #23 OR #24 OR #25 OR #26 OR #27 OR #28 OR #29 OR #30 OR #31 OR #32	1,321,109
#34	#4 AND #17 AND #33	7,567
#35	("Community Pharmacy Services"[Mesh] OR "Community Pharmacy Services"[tiab] OR "Services"[tiab])	135,550

#36	("Community Pharmacy Practice"[tiab] OR "Pharmacy Practice"[tiab] OR "Practice"[tiab])	289,005
#37	("Pharmaceutical Services"[Mesh] OR "Pharmaceutical Services"[tiab] OR "Pharmaceutical Care"[tiab])	26,898
#38	("Professional Pharmacy Services"[tiab] OR "Professional Practice"[Mesh] OR "Scope of Practice"[Mesh])	53,111
#39	("Evidence-Based Pharmacy Practice"[Mesh] OR "Patient-Centered"[tiab])	11,504
#40	("Health Care Facilities, Manpower, and Services"[Mesh] OR "Health Services Accessibility"[Mesh])	1,019,808
#41	"Health Care Quality, Access, and Evaluation"[Mesh]	3,104,734
#42	"Preventive Health Services"[Mesh]	207,650
#43	"Health Promotion"[Mesh]	37,363
#44	("Public Health"[Mesh] OR "Public Health"[tiab])	3,049,805
#45	#35 OR #36 OR #37 OR #38 OR #39 OR #40 OR #41 OR #42 OR #43 OR #44	3,693,620
#46	("Barrier"[tiab] OR "Barriers" OR "Obstacle" OR "Obstacles"[tiab])	122,679
#47	("Facilitator"[tiab] OR "Facilitators"[tiab])	11,979
#48	"Professional Practice Gaps"[Mesh]	207
#49	#46 OR #47 OR #48	126,806
#50	#4 AND #45 AND #49	1,392
#51	#34 OR #50	7,937
#52	#5 AND #51	66

Search of Medical Subject Headings terms are marked [Mesh]. Keywords in the title, abstract are marked [tiab].

Table 4. Search strategy using CINAHL. (continued)

#	Keyword/s or Search term/s	Results
S1	AB (("Community Pharmac*") OR (MM "Pharmacy, Retail" OR "Retail pharmac*") OR "Pharmac*")	9,512
S2	AB ((MM "Saudi Arabia") OR "Saudi Arabia" OR "Kingdom of Saudi Arabia")	290
S3	AB ("Public" OR (MM "Patients+") OR "Patient*" OR "Customer*" OR "Consumer*" OR "Client*" OR "Pharmacy User*" OR "User*" OR (MM "Pharmacists") OR "Community Pharmacist*" OR Policymaker* OR "Health Professional*" OR "Health Provider*" OR "Stakeholders")	175,989
S4	AB ("Knowledge" OR (MM "Knowledge") OR (MM "Professional Knowledge") OR (MM "Health Knowledge") OR (MM "Attitude to Health") OR (MM "Attitude of Health Personnel") OR (MM "Attitude") OR "Attitude*" OR (MM "Pharmacist Attitudes") OR (MM "Consumer Attitudes") OR (MM "Patient Attitudes") OR "Perception*" OR (MM "Perception") or "Perceived" OR "Behavior*" OR (MM "Behavior") OR "Behaviour*" OR "Awareness*" OR (MM "Cognition") OR "Opinion*" OR (MM "Public Opinion") OR "Perspective*" OR "View*" OR (MM "Patient Satisfaction") OR "Satisfaction" OR (MM "Consumer Participation") OR "Expectation*" OR "Experience*" OR (MM "Health Beliefs") OR "Belief*" OR "Contribution" OR (MM "Collaboration") OR "Collaboration" OR "Feedback")	153,769
S5	S1 AND S3 AND S4	2116
S6	AB ("Community Pharmacy Services" OR (MM "Pharmacy Service") OR "Pharmacy Service*" OR "Service*" OR "Community Pharmacy Practice" OR "Pharmacy Practice" OR "Practice" OR "Pharmaceutical Service*" OR "Pharmaceutical Care" OR "Professional Pharmacy Service*" OR "Professional Practice" OR "(MM "Practice Patterns") OR (MM "Professional Practice, Evidence-Based") OR " (MM "Patient Centered Care") OR (MM "Health Facilities") OR (MM "Health Services Accessibility") OR (MM "Quality of Health Care") OR (MM "Preventive Health Care") OR (MM "Health Promotion") OR (MM "Public Health"))	56,811
S7	AB ("Barrier*" OR "Obstacle*" OR "Facilitator*")	11,881
S8	S1 AND S6 AND S7	193
S9	S5 OR S8	2,193
S10	S2 AND S9	6

Search of CINAHL/Mesh Subject Headings terms using major concept are marked [MM]. Keywords in the abstract are marked [AB].

Table 4. Search strategy using Cochrane Library (continued)

#	Keyword/s or Search term/s	Results
1	"Community Pharmacy*" OR "Retail Pharmacy*"	743
2	"Saudi Arabia" OR "Kingdom of Saudi Arabia"	1412
3	"Public" OR "Patient*" OR "Customer*" OR "Consumer*" OR "Client*" OR "Pharmacy User*" OR "User*" OR "Pharmacists" OR "Community Pharmacist*" OR Policymaker* OR "Health Professional*" OR "Health Provider*" OR "Stakeholders	1142116
4	"Knowledge" OR "Attitude*" OR "Attitude to Health" OR "Attitude of Health Personnel" OR "Pharmacist Attitudes" OR "Consumer Attitudes" OR "Patient Attitudes" OR "Perception*" OR "Perceived" OR "Behavior*" OR "Behaviour*" OR "Awareness*" OR "Cognition" OR "Opinion*" OR "Public Opinion" OR "Perspective*" OR "View*" OR "Patient Satisfaction" OR "Satisfaction" OR "Consumer Participation" OR "Expectation*" OR "Experience*" OR "Health Beliefs" OR "Belief*" OR "Contribution" OR "Collaboration" OR "Feedback"	330823
5	"Community Pharmacy Services" OR "Pharmacy Service*" OR "Service*" OR "Community Pharmacy Practice" OR "Pharmacy Practice" OR "Practice" OR "Pharmaceutical Service*" OR "Pharmaceutical Care" OR "Professional Pharmacy Service*" OR "Professional Practice" OR "Practice Patterns" OR "Patient Centered Care" OR "Health Facilities" OR "Health Services Accessibility" OR "Quality of Health Care" OR "Preventive Health Care" OR "Health Promotion" OR "Public Health" OR "Barrier*" OR "Obstacle*" OR "Facilitator*"	169819
4	1 AND 2 3 AND 4 AND 5	1

Table 4. Search strategy using Ovid (continued)

#	Keyword/s or Search term/s	Results
1	"Community Pharmacy*" OR "Retail Pharmacy*"	3888
2	"Saudi Arabia" OR "Kingdom of Saudi Arabia"	20828
3	"Public" OR "Patient*" OR "Customer*" OR "Consumer*" OR "Client*" OR "Pharmacy User*" OR "User*" OR "Pharmacists" OR "Community Pharmacist*" OR Policymaker* OR "Health Professional*" OR "Health Provider*" OR "Stakeholders"	11130020
4	"Knowledge" OR "Attitude*" OR "Attitude to Health" OR "Attitude of Health Personnel" OR "Pharmacist Attitudes" OR "Consumer Attitudes" OR "Patient Attitudes" OR "Perception*" OR "Perceived" OR "Behavior*" OR "Behaviour*" OR "Awareness*" OR "Cognition" OR "Opinion*" OR "Public Opinion" OR "Perspective*" OR "View*" OR "Patient Satisfaction" OR "Satisfaction" OR "Consumer Participation" OR "Expectation*" OR "Experience*" OR "Health Beliefs" OR "Belief*" OR "Contribution" OR "Collaboration" OR "Feedback"	6294949
5	"Community Pharmacy Services" OR "Pharmacy Service*" OR "Service*" OR "Community Pharmacy Practice" OR "Pharmacy Practice" OR "Practice" OR "Pharmaceutical Service*" OR "Pharmaceutical Care" OR "Professional Pharmacy Service*" OR "Professional Practice" OR "Practice Patterns" OR "Patient Centered Care" OR "Health Facilities" OR "Health Services Accessibility" OR "Quality of Health Care" OR "Preventive Health Care" OR "Health Promotion" OR "Public Health" OR "Barrier*" OR "Obstacle*" OR "Facilitator*"	2272303
6	1 AND 2	24
7	3 AND 4 AND 5	510064
8	6 AND 7	45

Table 4. Search strategy using Google Scholar (continued)

#	Keyword/s or Search term/s	Results
#1	"Community Pharmacy" OR "Community Pharmacies" OR " Retail Pharmacy"	16,300
#2	"Community Pharmacy Services" OR "Pharmacy Services" OR "Pharmacy Practice" OR "Pharmaceutical Service" OR "Pharmaceutical Care" OR "Professional Pharmacy"	17,200
#3	"Saudi Arabia"	718,000
#4	#1 AND #2 AND #3	1000

Table 4. Search strategy using Scopus (continued)

#	Keyword/s or Search term/s	Results
1	"Community Pharmacy*" OR "Retail Pharmacy*"	153
2	"Saudi Arabia" OR "Kingdom of Saudi Arabia"	16500
3	"Public" OR "Patient*" OR "Customer*" OR "Consumer*" OR "Client*" OR "Pharmacy User*" OR "User*" OR "Pharmacists" OR "Community Pharmacist*" OR Policymaker* OR "Health Professional*" OR "Health Provider*" OR "Stakeholders"	18184
4	"Knowledge" OR "Attitude*" OR "Attitude to Health" OR "Attitude of Health Personnel" OR "Pharmacist Attitudes" OR "Consumer Attitudes" OR "Patient Attitudes" OR "Perception*" OR "Perceived" OR "Behavior*" OR "Behaviour*" OR "Awareness*" OR "Cognition" OR "Opinion*" OR "Public Opinion" OR "Perspective*" OR "View*" OR "Patient Satisfaction" OR "Satisfaction" OR "Consumer Participation" OR "Expectation*" OR "Experience*" OR "Health Beliefs" OR "Belief*" OR "Contribution" OR "Collaboration" OR "Feedback"	6859421
5	"Community Pharmacy Services" OR "Pharmacy Service*" OR "Service*" OR "Community Pharmacy Practice" OR "Pharmacy Practice" OR "Practice" OR "Pharmaceutical Service*" OR "Pharmaceutical Care" OR "Professional Pharmacy Service*" OR "Professional Practice" OR "Practice Patterns" OR "Patient Centered Care" OR "Health Facilities" OR "Health Services Accessibility" OR "Quality of Health Care" OR "Preventive Health Care" OR "Health Promotion" OR "Public Health" OR "Barrier*" OR "Obstacle*" OR "Facilitator*"	4541015
6	1 AND 2 AND 3 AND 4 AND 5	160

Table 4. Search strategy using the Web of Science (continued)

#	Keyword/s or Search term/s	Results
1	AB= (Community Pharmacy* OR Retail Pharmacy*)	5,539
2	AB= ("Saudi Arabia" OR "Kingdom of Saudi Arabia")	19,312
3	AB= ("Public" OR "Patient*" OR "Customer*" OR "Consumer*" OR "Client*" OR "Pharmacy User*" OR "User*" OR "Pharmacists" OR "Community Pharmacist*" OR Policymaker* OR "Health Professional*" OR "Health Provider*" OR "Stakeholders")	4,761,220
4	AB=("Knowledge" OR "Attitude*" OR "Attitude to Health" OR "Attitude of Health Personnel" OR "Pharmacist Attitudes" OR "Consumer Attitudes" OR "Patient Attitudes" OR "Perception*" OR "Perceived" OR "Behavior*" OR "Behaviour*" OR "Awareness*" OR "Cognition" OR "Opinion*" OR "Public Opinion" OR "Perspective*" OR "View*" OR "Patient Satisfaction" OR "Satisfaction" OR "Consumer Participation" OR "Expectation*" OR "Experience*" OR "Health Beliefs" OR "Belief*" OR "Contribution" OR "Collaboration" OR "Feedback")	5,391,225
5	AB=("Community Pharmacy Services" OR "Pharmacy Service*" OR "Service*" OR "Community Pharmacy Practice" OR "Pharmacy Practice" OR "Practice" OR "Pharmaceutical Service*" OR "Pharmaceutical Care" OR "Professional Pharmacy Service*" OR "Professional Practice" OR "Practice Patterns" OR "Patient Centered Care" OR "Health Facilities" OR "Health Services Accessibility" OR "Quality of Health Care" OR "Preventive Health Care" OR "Health Promotion" OR "Public Health" OR "Barrier*" OR "Obstacle*" OR "Facilitator*")	1,814,540
6	1 AND 2 AND 3 AND 4 AND 5	28

AB: Abstract. Last accessed date on 4 June 2021
