# Supplementary material for: Multi-stakeholder perspective on community pharmacy services in Saudi Arabia: A systematic review and meta-analyses for 2010–2020
Source: Explor Res Clin Soc Pharm. 2025 Apr 28;18:100608. doi: 10.1016/j.rcsop.2025.100608 (PMC12099458; doi:10.1016/j.rcsop.2025.100608)
Supplement: Supplementary material 3 — Summary of studies' characteristics and key findings. [file mmc3.pdf]

Supplemental material 3. Summary of studies' characteristics and key findings.

Abbreviations:

	M-Low: Moderate-Low | M-High: Moderate-High	ADEs: Adverse Drug Events.	GPs: General Practitioners.
N: Number of involved participants.	KAP: Knowledge, Attitude & Practice/Perception.	SM: Self-Medication.	DDIs: Drug-Drug Interactions.
Quan: Quantitative	CS: Cross-sectional.	MDIs: Metered Dose Inhalers.	VS: Vitamin Supplements.
Qual: Qualitative	SPV: Simulation Patient Visit.	GMs: Generic Medicines	HMs: Herbal Medicines.
MMAT: Mixed Methods Appraisal Tool.	ADRs: Adverse Drug Reactions.	CPs: Community pharmacists	NSAIDs: Non-Steroidal Anti-Inflammatory Drugs.

OTC and self-medication by community pharmacists

Reference/ Location	Aim	Method		Results	MMAT
		Study design	N		100%
Alkharfy, 2010<br>Riyadh City.	To assess the CPs' KAP towards HMs.	Quan.<br>CS. Survey	115	**Knowledge**: 12% reported it as excellent, 34% as very good, 35% as good & 19% as fair. 73%, knew about possible herb–Dis interactions<br>**Attitude**: 56%, expressed concerns about the safety of HMs. 53% believed that HMs are effective.<br>**Practice**: 100%, acknowledged dispensing HMs. 14% reported no known interactions and 13% were uncertain.<br>**Reasons** to dispense HMs: 49%, Vitality, 19%, Mental alertness, 17%, Insomnia, 5%, Anxiety & 1%, Low mood.	79%<br>M-High
Al-Rukban & Khalil, 2012<br>Riyadh City.	To investigate SM & adherence to OTC-directory.	Quan.<br>CS. Survey	405	**Knowledge**: 30.4% unaware of OTC-Directory.<br>**Malpractice:** the majority of CPs did not comply with its OTC-directory guidelines.	64%<br>M-Low
Al-Arifi, 2013<br>Riyadh City.	To assess the availability of HMs information resources.	Quan.<br>CS. Survey	1401	**Knowledge**: 87% were knowledgably about HMs.<br>**Attitude:** 20%, very often/often discuss HMs use with patients. 76%, were dispensing HMs. 30%, did not record.<br>**Barriers** to counsel patient: 46%, lack of time. 30.3%, lack of reliable sources. 15.2%, lack of scientific evidence to support the use of HMs. 13.4%, lack of HMs. knowledge. 9.1%, not interest in the subject.	71%<br>M-High
Bawazir, 2014<br>Riyadh City.	To assess oral healthcare.	Quan.<br>CS. Survey	141	**Knowledge**: Lack of scientific knowledge.<br>**Attitude**: Positive.<br>**Barriers**: lack of interaction between pharmacists and dental professionals.<br>**Malpractice***:* 13%, DAwP.	64%<br>M-Low
Alakhali, 2016b<br>Asir.	To evaluate SM pattern.	Quan.<br>CS. Survey	87	**Prevalence**: SM. is common among males (73.56%), females (26.43%) & adult group (67.81%).<br>**Reasons by CPs** for public SM: 67.8%, economic cost. 58.6%, time saving. 48.3% mild illness. 28.7%, no hospital/clinic nearby. 26.4%, quick relief. 25.3%, unwarranted intervention of GPs.	57%<br>M-Low
Al-Saleh et al., 2017<br>Eastern Region.	To assess oral healthcare.	Quan.<br>CS. Survey	279	**Knowledge**: 54% Lack of scientific knowledge.<br>**Attitude**: positive.<br>**Barriers**: 72, 4%, lack of interaction between pharmacists and dental professionals. 54.1%, ack of knowledge. 28%, lack of time. 28%, client not interested. 2.5%, lack of resources.	86%<br>H
Alsayari et al., 2018<br>Asir.	To evaluate the CPs' KAP towards HMs.	Quan.<br>CS. Survey	233	**Knowledge**: 84.1% was average of correct answers. 70% recognized possible side effects & DDIs.<br>**Attitude**: 79.4% agreed that HMs. is a CPs' responsibility.<br>**Practice**: 67.3% frequently dispense HMs. 36.5% frequently counsel clients about HMs. use.	86%<br>High
Ahmed & Khan, 2019<br>Al Kharj.	To assess the CPs' KAP towards HMs.	Quan.<br>CS. Survey	50	**Knowledge**: 76% have sufficient information about HMs.<br>**Attitude**: 90% believed that HMs are effective and 90% believed that HMs are safe.<br>**Practice**: 28%, always enquire about medical history. 38% always enquire about medication history. 48%, inform about potential Adverse effects.<br>**Barriers**: 62%, lack of time. 18%, lack of scientific evidence to support the use of HMs. 12%, lack of reliable sources. 10%, lack of HMs. knowledge. 4%, not interested in the subject.	64%<br>M-Low

OTC and self-medication by community pharmacists (continued)

Reference/ Location	Aim	Method		Results	MMAT
		Study design	N		100%
Ghosn et al., 2019<br>Al Khobar.	To assess KAP towards VS.	Quan.<br>CS. Survey	102	**Knowledge**: 42.2% know about VS-DDIs.<br>**Attitudes**: Positive practical approach.<br>**Practice**: 66.67% believed that consulting is their duty. 93.14% agreed that VS should dispense according to the prescription by nutritionists or physicians.	71%<br>M-High
Alhossan et al., 2019<br>Riyadh City.	To explore dispensing of proton pump inhibitors (PPIs).	Quan.<br>CS. Survey	1219	**Knowledge**: Most CPs know about PPIs indications & side effects.<br>**Attitude**: 94.3% dispense anti-ulcer drugs for their patients. 65.5% preferred to prescribe PPIs to their patients.<br>**Practice**: 32% reported ADEs. Reporting associated with CPs' experience. 76.7%, have never encountered any ADEs during the administration of PPIs.	71%<br>M-High
Aloudah et al., 2020<br>Riyadh City.	To assess OTC dispensing.	Mixed Methods<br><br>SPV.<br>Focus groups	 <br>214<br>13	Low adherence to EBPs. Only 40% of CPs dispensed OTC according to EBPs<br>**Barriers**: Conflicts between available guidelines & health patient literacy.<br>**Facilitator**: Establishing patient-pharmacist relationships to facilitate EBP.	71%<br>M-High
Kurban et al., 2020<br>Jeddah.	To evaluate the awareness level towards sunscreens & moisturizers products.	Quan.<br>CS. Survey	310	The majority of CPs indicated that they need more knowledge about cosmetics.<br>53% were aware about the sunscreen, while 62% have the knowledge about moisturizers.	57%<br>M-Low
S. M. Alshahrani, 2020<br>Asir.	To investigate the CP' KAP towards Weight Reduction Products (WRPs).	Quan.<br>CS. Survey	191	**Knowledge**: 50% provided incorrect answers. 85% do not know which the US FDA are approved OTC products.<br>**Attitude**: 58.68% believed that WRPs are effective & safe.<br>**Practice:** 75.39% dispense WRPs without prescription.	93%<br>High
A. Alshahrani, 2020a<br>Nationwide.	To determine the CPs' KAP towards VS. counselling.	Quan.<br>CS. Survey	1041	**Knowledge**: CPs have adequate knowledge about VS & nutritional supplement.<br>**Attitude**: Positive.<br>**Practice**: 81.84%, frequently counsel clients about VS. side effects. 66.72%, agreed to conducting follow-ups	79%<br>M-High

OTC and self-medication by public.

Reference/ Location	Aim	Method		Results	MMAT
		Study design	N		100%
Suleiman, 2013<br>Riyadh City.	To assess SM by patients with OTC.	Quan.<br>CS. Survey	537	**Prevalence:** 67.2% practice SM. 63% were male.	93%<br>High
Aljadhey et al., 2015<br>Riyadh City.	To estimate SM prevalence	Quan.<br>CS. Survey	538	**Prevalence:** 52.97% practice SM (73% were male), 51.2% with OTC while 48.8% with POMs.<br>**Knowledge**: Poor knowledge. 68.2%, do not distinguish between POM & OTC. 55.5%, did not tell about other used medications<br>**Attitude**: 62.6% had positive attitude.<br>**Malpractice**: 285 medications were purchased. Of these, 139 (48.8%) POMs (22% Antibiotics).<br>**Reasons**: Time saving and minor illnesses.	86%<br>High
Ansari et al., 2020<br>Hail.	To determine the factors towards SM.	Quan.<br>CS. Survey	663	**Prevalence**: 90.2%. Public with low education level, non-healthcare professionals and higher income were more likely to prefer SM.<br>**Perceptions**: 33.5% agreed that SM is a safe practice.<br>**Reasons**: Minor Illnesses were (90.2%), time saving (82.2%), convenient (74.7%), quicker relief (66.1%), and economical (61.2%).	100%<br>High
K. A. Khan et al., 2020<br>Jeddah	To determine the prevalence & factors of SM.	Quan.<br>CS. Survey	1036	**Prevalence**: SM. practice is prevalent**.**<br>**Knowledge:** 72.68 know that some medications lead to reactions. 63.32% know that some medications lead to DDIs.<br>**Reasons**: 67.18 % due to minor health issues, 58.01% knowledge about medications and diseases. 33.1% are seeking quick relief. 28.28% avoided long time waiting in hospitals. 16.69% high cost of medical consultation.	71%<br>M-High

Dispensing generic medicines by community pharmacists.

Reference/ Location	Aim	Method		Results	MMAT
		Study design	N		100%
Wajid et al., 2015<br>Riyadh City.	To evaluate the CPs' knowledge & perception towards dispensing GMs.	Quan.<br>CS. Survey	365	**Knowledge**: Lack of basic information on GMs. 62.5% have doubts on therapeutic efficacy & safety profiles.<br>**Perception**: 60.6% supported GMs. CPs reported the need for guideline (78.9%) & collaboration with GPs. (80.5%).<br>**Factors** for GMs dispensing: 74.3%, Patient's socio-economic. 61.1%, Credibility of manufacturers/supplier. 60.3%, Patients demands. 59.1%, Product bonus offered by pharmaceutical companies.	86%<br>High
Albadr and Khan, 2015<br>Al Ahsa.	To explore the factors towards GMs dispensing.	Qual.<br>Interview	20	Customer' appearance is affecting the CPs decision to dispense GM.<br>Pharmacists in community hospital influenced by GPs' recommendation for branded medicines.	71%<br>M-High
Alkhuzaee et al., 2016<br>Makkah Region.	To assess the CPs' knowledge & perception towards GMs.	Quan.<br>CS. Survey	121	**Knowledge**: Deficits about GMs & their substitution.<br>**Perception**: 68.5% supported GMs. 79% dispensed GMS. 60.3% reported the need of information on safety & efficacy.<br>**Factors** for GMs dispensing: 66.7%, Cost-saving. 53.3%, Availability of stock. 58.3%, Patient's demand. 35%, Having no other choice. 19.2%, Patient's appearance.	79%<br>M-High

Dispensing prescription only medicines (POMs) by community pharmacists.

Reference/ Location	Aim	Method		Results	MMAT
		Study design	N		100%
Khan and Ibrahim, 2013<br>Al Hafof.	To investigate non-prescription dispensing & ADEs reporting.	Qual.<br>Interview	20	**Malpractice**: DAwP is common practice.<br><br>**ADEs reporting**: Lack of awareness among CPs of the process.	71%<br>M-High
Al-Mohamadi et al., 2013<br>Jeddah.	To investigate the CPs' practice toward dispensing POM.	Quan.<br>SPV.	60	**Malpractice**: CPs are violating the law. In 119 requests, 97.9%, DAwPs, 100%, dispensed captopril & 89.5% gave the antipsychotic.<br>**Reasons**: 20%, If I did not, other CP would dispense. 16.3%, no such thing as OTC list, 14.13%, has some authority to dispense, 13.59%, No fine or control by the SFDA. 11.96%, the drug dispensed is not harmful, 11.96%, there is no policy prohibit dispensing without prescription.	57%<br>M-Low
Al-rukban and Rizvi, 2014<br>Riyadh City.	To investigate written prescription dispensing.	Quan.<br>CS. Survey	405	**Knowledge**: 28.9% never attended any CME activity.<br>**Practice**: asked before dispensing about symptom (92%), allergy (60%) & pregnancy (90%). 53%, refer to the GPs CPs informed patient about drug side effects (35.8%), about DDIs (45.7%), duration (73%) & Provided possible ADRs, warnings & precautions (41%).<br>**Reasons** by CPs to dispense without prescription: 57.6%, Patients' request. 51.1%, CP' long experience. 46.3%, CP' knowledge & background. 37.7%, treating previous similar cases. 23.6%, minor symptoms.	57%<br>M-Low
Alrwisan et al., 2014<br>City of Riyadh,<br>Buraydah & Hail.	To explore the CPs' dispensing oral isotretinoin-products.	Quan.<br>CS. Survey	160	**Knowledge**: CPs unaware of the proper use & risks associated with this product for pregnant women.<br>11% did not ask about a pregnancy test prior to dispensing.<br>**Malpractice**: 20% dispensing POMs without prescription.	71%<br>M-High
Khan and Emeka, 2015<br>Al Ahsa.	To assess the CPs' views about the non-prescription medication for citalopram	Qual.<br>Interview	11	**Malpractice:** Non-prescription sale is a frequent practice.	71%<br>M-High
Suleiman et al., 2016<br>Al Ahsa.	To investigate request for POMs of aphrodisiacs products.	Quan.<br>CS. Survey	53	**Malpractice**: The non-prescription dispensing of POMs including sexual enhancement is common practice.	57%<br>M-Low
Bahnassi, 2016<br>Riyadh City & Jeddah.	To investigate the dispensing POMs. without prescription	Quan.<br>CS. Survey	58	**Malpractice**: 93% admitted dispensing DAwP & POMs without prescription.<br>**Reasons**: 89%, patients' socioeconomic status, 71%, patients' demand. 68%, minor symptoms. 45%, if not dispensing, patients easily can get it from other pharmacy. 27%, increasing sales.	71%<br>M-High
Alshammari et al., 2017<br>Riyadh, Jeddah, Madinah, Hail, Al Qassim & E. Region.	To assess the CPs' compliance with the law of POMs. dispensing.	Quan.<br>SPV.	150	**Malpractice**: Non-compliance of CP' practices with the law.<br>63% approved dispensing POMs with a high-risk profile of ADRs without a valid prescription.	57%<br>M-Low

Dispensing prescription only medicines (POMs) by public.

Reference/ Location	Aim	Method		Results	MMAT
		Study design	N		100%
Alotaibi and Abdelkarim, 2015<br>Al Dawadmi.	To explore consumer's perceptions towards the role of CPs in dispensing.	Quan.<br>CS. Survey	100	**Perception**: The professional performance of CPs is below expectation.<br><br>72% perceived that CPs violate the law by **dispensing** POM without prescription.<br>26%, encountered a dispensing error.<br>48% do not receive enough **counselling**. Consumers are embarrassed to ask CPs and need privet area (94%).	57%<br>M-Low
Alosaimi et al., 2016<br>Riyadh City.	To explore the causes behind psychotropic without a prescription.	Quan.<br>CS. Survey	302	**Malpractice**: Easily obtain psychotropic medications without a prescription. Prevalence of POMs 80.20%<br>**Reasons**: Non-serious symptoms (42.4%) & high cost of psychiatric services (28.5%), Lack of trust in psychiatrists or lack of time (22.50%). Unavailability of medical services near residence (19.20%)	93%<br>High

Dispensing antibiotic without prescription (DAwP) by community pharmacists.

Reference/ Location	Aim	Method		Results	MMAT
		**Study design**	**N**		**100%**
Bin Abdulhak et al., 2011<br>Riyadh City.	To determine the DAwP. By CPs.	Quan.<br>SPV.	327	**Malpractice**: 77.60% DAwP. None of CPs asked about antibiotic allergy or provided information about DDIs. Only 23%, who dispensed antibiotics inquired about pregnancy status.	71%<br>M-High
Emeka et al., 2012<br>Al Ahsa.	To identify the CPs' attitude & practice towards DAwP.	Qual.<br>Interview	15	**Attitude**: Negative.<br>**Malpractice**: Significant incidence of misuse regarding DAwP.	57%<br>M-Low
Hadi et al., 2016<br>Makkah Region.	To evaluate the CPs' KAP towards DAwP.	Quan.<br>CS. Survey	189	**Attitudes**: 78.3% reported they should stop DAwP. 76.9%, always asked the patient about drug allergies. 70.4% ask about concomitant drugs or comorbidities. 88.9% inform about potential side effects. 81% ask about other medication.<br>**Malpractice**: 70.5%, not aware that DawP is illegal practice. 55%, believed that DawP is common practice.<br>**Reasons** by CPs to DawP.: 69.9 patients don not want to visit GPs because of minor symptoms. 65.3%, patients cannot afford to consult GPs. 45.8%, CPs have good knowledge about antibiotic. 26.8%, increased sales. 28.4%, lack of awareness about rules and regulations.	71%<br>M-High
Al Akhali, 2016a<br>Asir.	To explore the CPs' KAP towards antibiotic use.	Quan.<br>CS. Survey	120	**Attitude**: 36% believe that these antibiotics are not harmful. 29%, claimed that there is no control by SFDA. 61.60% considered self-medication with antibiotics accepted practice.<br>**Malpractice**: 15.8% admitted DAwP. 19.10%, before dispensing did not ask about any medications taken. 27.5%, don't inform about DDIs.<br>**Reasons**.: 52%, patients' confidence in CPs. 41%, patients' economic status. 5% drug refilling. 2%, patient did not get benefit of previous medicines.	57%<br>M-Low
M. Mahmoud et al., 2018<br>Riyadh City.	To explore the reasons behind DAwP.	Qual.<br>Interview	16	**Malpractice**: DAwP is common practice.<br>**Reasons**: Public demand. Financial considerations; pharmaceutical companies & pharmacy management	79%<br>M-High
Alhomoud et al., 2018<br>Eastern Region.	To explore the reasons behind DAwP.	Qual.<br>Interview	20	**Malpractice**: DAwP is common practice.<br>**Reasons**: Availability of pharmacies, CPs need for business survival, inappropriate practices of the public, customer pressure & CPs & patients' trust.	86%<br>High
Alrasheedy et al., 2020<br>Al Qassim.	To evaluate the impact of law enforcement on DAwP.	Quan.<br>CS. Survey<br>SPV.	116<br>116	Law enforcement was effective. The prevalence of DAwP significantly decreased by 80%.<br>**CS finding:** Before the law enforcement (2017): 70.7%, believed that DawP is common practice while 12.9%, after law enforcement (2018).<br>**SPVs finding:** DawP average before the law enforcement (92.15%). DawP average after law enforcement (8.65%) Improvement in counselling in the post-law enforcement period compared to the pre-enforcement phase regarding the importance of adherence & appropriate use of antibiotics.<br>**Malpractice**: DawP is still present.	71%<br>M-High
Al-Tannir et al., 2020<br>Riyadh City.	To assess the rate of DAwP sales & compare it with the 2011 study.	Quan.<br>SPV.	327	DAwP decreased 77.6% compared to 2011.<br>**Malpractice**: DAwP still occurs. 12.5% of CPs are DAwP. Only 3.9% asked about drug allergies. 33.3% provided information about DDIs.<br>None of them asked about pregnancy status.	64%<br>M-Low
Alrukban et al., 2020<br>Riyadh City.	To explore DAwP law.	Quan.<br>CS. Survey	268	Law enforcement was effective. DAwP have seen a 16.6% drop in 2019 compared to 2017.<br>24.83%, believed that DAwP is common practice even after the enforcement of the law.<br>41.26% agreed that law enforcement negatively impacted the pharmacy profits.	93%<br>High

Counselling practice by community pharmacists.

Reference/ Location	Aim	Method		Results	MMAT
		Study design	N		100%
Al Hassan, 2010<br>Riyadh City.	To investigate patient counselling.	Quan.<br>CS. Survey	114	**Attitude**: Positive.<br>**Barriers to counsel**: CPs agreed/strongly agreed on: Lack of feedback from patients (31.58%). Don't know about drug and its effect (29.82%). Lack of patient medical history (26.32%). Lack confidence in their knowledge (22.81%). Concerns about contradicting GPs (21.05%). Lack of time (17.54%). customers don't perceive benefit (17,54%), don't know how to approach people (09,65%).	64%<br>M-Low
Alaqeel and Abanmy, 2015<br>Riyadh City.	To investigate counselling practices.<br>To determine POMs dispensing.	Quan.<br>CS. Survey<br>SPV.	350<br>150	**161 SPVs**: Deficiencies in appropriate medication counselling. CPs inquired about medications history (08.00%), concomitant drugs used (09.30%), or drug allergy (07.30%). Gave possible ADRs, warnings & precautions (10.00%). Duration (13.3%).<br>**Survey**: Asking about Possible side effects always (7.4%), usually (9.4%). Gave information about DDIs always (13.4%), usually (21.1%). Food-DIs always (15.7%) usually (22.5%). Availability of GMs always (16.2%) usually (24.0%). Storage conditions always (26.6%) usually (22.5%). Satisfied with counselling practice always (37.1%) usually (48.2%).<br>**Malpractice**: CPs dispensed POMs as well as DAwPs.<br>**Barriers**: CPs agreed/strongly agreed on: lack of patient medical history (61.90%), lack of time (59.60%), limited drug information resources (38.50%) & lack confidence in their knowledge (24.80%).	71%<br>M-High
Kashour et al., 2016<br>Riyadh City & Jeddah.	To evaluate the quality of counselling<br>To determine POMs dispensing.	Quan.<br>SPV.	600	Counselling qualities are suboptimal in acute cardiac conditions<br>**Malpractice**: 63.2% dispense POM to simulated cardiac patients without prescription.	71%<br>M-High
Alfadl et al., 2018<br>Al Qassim.	To evaluate counselling skills & content.	Quan.	11	The observational study revealed that overall standard of counselling was poor.	64%<br>M-L
Al Qarni et al., 2020<br>Bisha.	To evaluate counselling practice.	Quan.<br>SPV.	90	Patient counselling provided by CPs was suboptimal**.** CPs inquired about medications history (52.40%), concomitant drugs used (0%), or drug allergy (02.70%), Duration (23.8%). 100% did not give possible ADRs, warnings & precautions.<br>**Malpractice**: 85% (62 out of 73 SPVs.) did not ask for physician prescription to dispense POMs.	71%<br>M-High
A. Mahmoud et al., 2018<br>Makkah Region.	To investigate acute back pain consultations.	Quan.<br>SPV.	300	Consultations were inadequate. CPs did not ask about other diseases (91%), any medications taken (90%) & drug allergies (99%). 98% did not give information about expected drug problems.<br>Counselling time was less than one minute in 67.67% of the cases.	71%<br>M-High
Khojah, 2019a<br>Al Madinah	To investigate sedating antihistamines consultations.	Quan.<br>SPV.	88	Insufficiency in the quality of counselling. 26.1% of CPs offered spontaneous counselling. Of them, 95% mentioned sedation as side effect. 73.9% of CPs offered counselling upon customer request. Of them, 66% mentioned sedation as side effects.	71%<br>M-High
Khojah, 2019b<br>Al Madinah.	To investigate privacy during counselling**.**	Quan.<br>SPV.	80	Physical measures of privacy were unavailable in all community pharmacies.<br>In all pharmacies, counseling was offered at the general dispensing and cashier counter in the presence of other customers nearby.	57%<br>M-Low

Counselling on asthma & MDIs use by community pharmacists.

Reference/ Location	Aim	Method<br>Study design	N	Results	MMAT<br>100%
Khan and Azhar, 2013<br>Al Ahsa.	To assess the CPs' skills towards MDIs use.	Quan.<br>SPV.	71	Poor recognition of 7 MDIs. steps. (Average = 42%).<br>Young CPs in chain pharmacies have better skills than others.	64%<br>M-Low
Alqahtani et al., 2015<br>Riyadh City.	To assess competency on MDIs use & factors affecting competency.	Quan.<br>SPV.	164	Poor knowledge of 7 MDIs. steps. (Average = 42%). Only 7.3% performed correct steps.<br>Experience (>5 years), age (>29) & CPs in chain have better skills.	57%<br>M-Low
Adnan et al., 2015<br>Al Qassim.	To assess the competency on MDIs use & compare it with (Khan and Azhar, 2013)	Quan.<br>SPV.	96	Poor recognition of MDIs. steps. (Average = 49%). Only 7.2% performed correct steps.<br>No significant difference between the two studies.	57%<br>M-Low
Alotaibi et al., 2016<br>Al Dawadmi.	To evaluate the CPs' contribution in asthma.	Quan.<br>CS. Survey	20	Most CPs are competitive enough in counselling asthma patients.<br>**Barriers**: 80% lack time while 15% claimed they are underpaid to provide counselling.	57%<br>M-Low
Khojah and Abdalla, 2019<br>Al Madinah.	To investigate the counselling for non-buyers.	Quan.<br>SPV.	80	77% gave certain information although they were aware that the client was not having the intention to buy the medication:<br>23%, refused to give any information & requested the client either refer to GPs, refer to the pamphlet, or to search for the instructions online.<br>MDIs: 5.3%, gave the full instructions. 68% gave fair instructions & 4% gave insufficient instructions.	57%<br>M-Low

Community pharmacy users' relevant counselling attributes.

Reference/ Location	Aim	Method<br>Study design	N	Results	MMAT<br>100%
Al Aqeel & Hiligsmann, 2018<br>Riyadh City.	To identify community pharmacy users' relevant counselling attributes.	Mixed Methods,<br>CS. Survey	120	Most **attributes** were:<br>The quality of the information during counselling.<br>The CPs' experience & education.<br>Explanation of side effects & DDIs.	71%<br>M-High

Interventional clinical program by CPs to counsel people with diabetes.

Reference/ Location	Aim	Method<br>Study design	N	Results	MMAT<br>100%
(Y. Khan et al., 2020)<br>Riyadh, Jeddah, Tabuk, Al Dammam, Al Hafof, Abha & Al Maddinah.	To evaluate the outcomes of clinical program by CPs to counsel people with diabetes.	Quan.<br><br>Pharmacists<br>Patients	 <br>20<br>1137	A pharmacy-led diabetes education program is feasible.<br>Improvements in diabetes awareness, attitudes towards diabetes and medication adherence	93%<br>High

Medication Safety measures by community pharmacists.

Reference/ Location	Aim	Method		Results	MMAT
		Study design	N		100%
Al-Arifi, 2014a Riyadh City.	To assess the CPs' attitudes toward dispensing errors.	Quan. CS. Survey	656	**Attitudes**: CPs are aware of DEs. **Top factors enhancing DEs**: 68.6%, look-alike sound-alike drugs. 66%, fatigue. 65.7%, existence of GMs. 62.7%, lack of privacy when dispensing. 59.9%, interruption. 58.2%, noise. 58%, insufficient technical resources. 57.8%, job dissatisfaction. 54.4%, design of dispensary and layout of shelves. 54.1%, design of computer dispensing software. 48.6%, package labelling	71% M-High
Al-Arifi, 2014b Riyadh City.	To identify ethical issues with CPs.	Quan. CS. Survey	682	**Knowledge**: Lack of knowledge of ethical issues. **Barriers** to discuss ethical issues: Lack of time (69.2%). Lack of reliable resources (10.7%). Not interested in subject (10.1%) Lack of ethical knowledge (4.8%).	71% M-High
Alrabiah et al., 2017 Riyadh City.	To evaluate the medication safety during pregnancy.	Quan. CS. Survey	256	**Knowledge**: CPs have an inadequate knowledge. **Practice**: CPs do not always offer correct advice to pregnant women. 54.7% asked female patients about the pregnancy status.	71% M-High
Kamel et al., 2018 Jeddah.	To investigate the prevalence of errors in prescription writing.	Quan. Descriptive Study	12	Prescribing Errors are common among GPs' prescription. The quality of prescription writing was deficient in some elements.	71% M-High
Alrabiah et al., 2019 Riyadh City.	To evaluate the potential common DDIs.	Quan. CS. Survey	283	**Knowledge**: was inadequate. Among the 26 drug pairs, only five of them were identified correctly. The lack of electronic systems has a serious impact on patient safety.	79% M-High
Alrefaei et al., 2019 Nationwide.	To assess the CPs' KAP toward Antimicrobial Stewardship (AMS).	Quan. CS. Survey	500	AMS program practice was poor.	79% M-High
Mobrad et al., 2020 Riyadh City.	To assess drug abuse & misuse.	Quan. CS. Survey	239	13.59% CPs reported complete absence of authorities in monitoring their services. 94.6%, claimed that they advise their patients about ADRs. 36.4% of CPs believed that administrating controlled medications without a prescription is a necessary for income.	71% M-High
A. Alshahrani, 2020b Nationwide.	To investigate the CPs' readiness to COVID-19.	Quan. CS. Survey	1097	**Readiness:** Pharmacy providing hand sanitizer for customers: 93.3% always, 3.6% very often, 2.6% sometimes while 0.2% never provided. 83.8% of the pharmacies had self-protective windows while 16.2% did not have. **Preparedness:** Results were significant to authoritatively point out that CPs were prepared to play a supportive role	86% High
Khojah, 2020 Al Madinah.	To investigate the CPs' preventive measures against to COVID-19	Quan. SPV.	100	Poor compliance with preventive measures against COVID-19. Unsatisfactory level of preparedness for COVID-19.	57% M-Low
Malebari et al., 2020 Jeddah.	To assess safety information towards NSAIDs.	Quan. CS. Survey	200	Insufficiencies in counselling practice. CPs screened risk factors regularly for selective (51.90%) and non-selective (54.80%) NSAIDs. CPs gave advice to prevent ADRs regularly for selective (50.10%) and non-selective (52.50%) NSAIDs. CPs sought information about concomitant drugs regularly for selective (40.50%) and non-selective (42.50%) NSAIDs.	86% High

Patient safety measures by multi-stakeholders

Reference/ Location	Aim	Method		Results	MMAT
		Study design	N		100%
Al Juffali et al., 2019a<br>Riyadh City.	To explore the safety problems associated with medication supply from community pharmacies.	Qual.<br><br>Interview<br>Focus groups	 <br>35	**Users group**: Need medication information from CP as healthcare professionals & not as a vendor.<br>**CPs group**: Some of them perceived the CPs as salesmen, while others perceived them as professionals.<br>**Professionals group**: Perceived CPs could play a role in caring for patients.<br>**Factors** contribute to medication safety problems: Commercial pressures on CPs by pharmaceutical companies.<br>A failure to enforce regulations, the fragmented healthcare system (control, regulation & lack of patient database) & SM.	100%<br>High
Al Juffali et al., 2019b<br>Nationwide	To derive priorities of medication safety problems	Quan.<br>CS. Survey	 166	The top five **priorities** were: Lack of pharmacy facilities (i.e. counselling area), lack of communication between CPs & GPs, lack of patient databases, lack of post-registration CPs education, and long working hours.	93%<br>High
AlShayban et al., 2020<br>Al Khobar.	To evaluate the association between patient's knowledge & medication adherence.	Quan.<br>CS. Survey	 318	33% had high adherence with type 2 diabetes. Half of patients had disease knowledge between 51% - 75%.<br>Patients with better knowledge were more to have high adherence.	100%<br>High

Patient Safety measures (ADRs reporting by community pharmacists).

Reference/ Location	Aim	Method		Results	MMAT
		Study design	N		100%
Khan, 2013<br>Al Ahsa.	To analyse barriers to ADRs reporting.	Quan.<br>CS. Survey	50	**Knowledge**: Poor understanding of the reporting system process.<br>**Barriers**: 88% of reporting forms are not available. 86%, unavailability of professional environment to discuss ADRs. 44% do not know how to report. 38%, CPs not motivated to report. 34%, reporting is time consuming. 18%, reporting forms are too complicated. 12%, fear of legal liability of the reported ADRs. 2%, confidence whether it is an ADRs / insufficient knowledge of pharmacotherapy in detecting ADRs.	71%<br>M-High
Al-Hazmi and IL, 2013<br>Makkah City.	To evaluate ADRs reporting.	Quan.<br>CS. Survey	170	**Knowledge**: 82.35%, not aware of ADRs reporting system.<br>**Barriers**: 59%, forms were not available. 52% believed that they may not care about it. 41.17% believed that all ADRs had been detected before marketed drug. 29% did not know which address to send the reports. 23%, reporting forms are too complicated. 23%, reporting was too time consuming. 17.6%, had insufficient clinical knowledge about ADR reporting.	50%<br>Low
Mahmoud et al., 2014<br>Riyadh City.	To assess ADRs reporting.	Quan.<br>CS. Survey	104	**Knowledge**: 77.9% have poor knowledge about ADRs reporting.<br>**Attitude**: 16.6%, believe that ADRs reporting is a duty of GPs. 87.5% did not report ADRs. 12.5% said they reported ADRs when they occurred. 24%, never discussed ADRs with GPs. 50%, always counsel patients on ADRs. 44%, always discuss medication allergy. 72%, asking female about pregnancy.	64%<br>M-Low
Rabba and Ain, 2015<br>Al Kharj.	To evaluate ADRs reporting.	Quan.<br>CS. Survey	53	**Knowledge**: 70%, do not know about ADRs reporting system. 74%, do not know where to report the ADRs. 79%, never reported ADRs.<br>**Barriers**: 91%, forms are not available, 79%, confidentiality. 74%, Causality uncertain. 70%, reporting address is unknown. 70%, patient's confidence. 60%, No motivation. 55%, Fear of liability. 55%, Do not know how to report. 42%, reporting is time consuming. 38%, Insufficient clinical knowledge. 30%, complicated forms.	71%<br>M-High
Ali et al., 2020<br>Al Dammam.	To assess barriers towards ADRs reporting.	Quan.<br>CS. Survey	101	**Knowledge**: 50% of CPs claimed that they know about ADRs reporting system.<br>**Barriers**: 47.52%, professional obligation. 33.66%, Haven't time to report. 33.66%, Unavailability of professional environment to discuss ADRs. 29.7%, Reporting is time consuming. 26.73%, Insufficient knowledge of pharmacotherapy in detecting ADRs. 26.68% of forms are not available. 26.68%, fear of legal repercussions. 23.76%, No actions taken based on reporting. 23.76%, forms are too complicated. 23.76% fear legal liability. 21.78%, not confident whether it is an ADRs. 20.79%, not motivated to report	86%<br>High

Patient safety measures (ADRs reporting by public).

Reference/ Location	Aim	Method		Results	MMAT
		Study design	N		100%
Almubark et al., 2020<br>Nationwide.	To estimate the prevalence & awareness of ADRs reporting.	Quan.<br>CS. Survey	5228	**Prevalence**: 28% experienced ADRs over a year. Gastrointestinal disorders were the most reported.<br>**Awareness**: who experienced ADRs, 30.26% were aware of reporting system but 14.29% filed to report.	93%<br>High

Clinical services by community pharmacists.

Reference/ Location	Aim	Method		Results	MMAT
		Study design	N		100%
Alahdal et al., 2016 Jeddah.	To evaluate CPs services.	Quan. CS. Survey	99	Many important services were lacking & did not meet the international standards. **Malpractice**: 29% dispense prescribed drugs without prescriptions. 29% unaware of ADRs reporting. **Barriers** to counsel patients**:** 60%, patient culture & education. 40%, lack of time. 9%, physical barrier. 15%, shortage of staff. 15%, language barriers.	50% Low
Ahmed and AL-Wahibi, 2016 Al Qassim.	To assess the CPs' KAP towards services.	Quan. CS. Survey	224	**Knowledge**: 52.7% think that they are knowledgeable about PC. **Barriers**: 86.8%, workload, 50%, lack of clinical knowledge, 49.6%, staff shortage, 47.8%, lack of PC technical knowledge. 29%, lack of information sources. 41.1%, lack of knowledge on how to reach the information, 23.2%, Physical, 21.8%, lack of communication with patients, 19.6%, lack of patient's time/demand, 17.4%, Lack of communication with physicians.	57% M-Low
Gillani et al., 2017 City of Riyadh, Jeddah, Al Madinah & Makkah.	To assess clinical services.	Quan. CS. Survey	1258	Effective clinical services inbound with CPs' knowledge, need, value & confidence. 66%, denied practicing clinical services. 64%, highlighted less performance to their respective potential.	86% High
Balkhi et al., 2018 Riyadh City.	To assess the CPs' readiness to provide an immunization service.	Quan. CS. Survey	139	CPs who were ready & willing to provide immunization services (55%) **Barriers:** 75.4%, Lack of training. 67.4%, Concerns in patient safety. 55.6%, lack of time. 53.7%, adding more workload. CPs are less trusted by customers. 51.1%, Conflicts with other professionals. 44.9%, Concerns about handling vaccines, storage, and disposal of sharps. 37.5% of CPs are not comfortable using needles.	71% M-High

Clinical services from public perspectives.

Reference/ Location	Aim	Method		Results	MMAT
		Study design	N		100%
Almansour et al., 2020 City of Riyadh & Najran.	To explore the consumers' perceptions about current & future services of CVD management.	Qual. Interview	25	They perceived CPs' role as medication supply with low trust in CPs. Pharmacy-based services not provided by CPs. Participants willing to engage in future pharmacy delivered preventive health services.	86% High
Alzayer et al., 2020 Riyadh City.	To explore perspectives of asthmatic patients about using pharmacy-based services.	Qual. Interview	23	Perspectives were unclear about pharmacy asthma care services. Participants reported not having experienced such services. Few participants believed that pharmacists have sufficient medical knowledge to be able to educate them about asthma	71% M-High
Al Aloola et al., 2020 Riyadh City.	To explore community's needs & preferences related to immunization services.	Qual. Interview	20	Lack of knowledge about immunization service. Participants feel confidence with being immunized by CPs & few accept immunization if CPs had received proper training. A need for community pharmacy immunization services with **concerns**: Lack of private area to conduct the service & female CPs. The cost of the immunization service.	86% High

Clinical services from multi-stakeholders' perspectives

Reference/ Location	Aim	Method		Results	MMAT
		Study design	N		100%
Mazhar et al., 2017)<br>Al Dhahran &<br>Al Dammam.	To describe the inter-professional collaboration between GPs & CPs concerning PC services	Qual.<br>Interview<br>CPs & GPs	54	Lacking in professional trust social acceptance:<br>**GPs**: Not yet aware of the role of CPs.<br>**CPs**: would like to have more relationship with GPs.<br>**Barriers**: Lack of knowledge about CPs-derived PC services & trust.<br>**Facilitators**: Enhancing collaboration between CPS & GPs by integrating the PC model with the health system.	57%<br>M-Low
Alhaddad, 2019<br>Makkah Region.	To measure youth perceptions towards Medication Therapy Management (MTM).	Quan.<br>CS. Survey	953	96% perceived MTM program to be beneficial for patients' care. 72.4% believed that CPs are qualified to provide MTM services. 70.3%, willing to register for the MTM program if implemented.	86%<br>High
Rasheed et al., 2020<br>Al-Qassim,<br>Riyadh City & Jeddah.	To identify stakeholder's views about the extended role of community pharmacy.	Qual.<br>Interview	15	**The stakeholder's views**: -<br>The current practice is product oriented rather than actual PC practices.<br>CPs can deliver patient-centered care services if encouraged.<br>**Facilitators**:<br>Updating new regulations regarding patient-centered care services.<br>Enhancing CPs' clinical knowledge & skills & CPs need to offer specialized services.<br>**Motivators**:<br>MTM, clinical monitoring & medication adherence can be the motivation to change practice.<br>Effective collaboration between authorities is necessary for the successful transition of practice.	86%<br>High

Job satisfaction by community pharmacists.

Reference/ Location	Aim	Method		Results	MMAT
		Study design	N		100%
Suleiman, 2015<br>Riyadh City.	To measure job satisfaction among CPs.	Quan.<br>CS. Survey	394	Independent CPs had lower satisfaction levels.<br>**Job stressors**: long working hours, difficulty in obtaining casual or sick leave, and low salaries.	79%<br>M-High
Zahrani et al., 2017<br>Eastern Region.	To highlight the factors behind less job satisfaction of CPs.	Quan.<br>CS. Survey	103	CPs expressed displeasure regarding duration of work (52.4%), working shift (37.9%) and weekend-break (50.5%). CPs seemed satisfied regarding other allowances such as salary (54.4%), overtime (41.7%), bonus (39.8%), vacations (45.6%), tickets (65%), housing allowance (33%) and health insurance (48.5%).<br>**Factors**: Workload & duty shifts were unsatisfying.	86%<br>High

Service satisfaction by public.

Reference/ Location	Aim	Method		Results	MMAT 100%
		Study design	N		
Al-Arifi, 2012<br>Riyadh City.	To assess patients' perception & satisfaction with CPs' roles.	Quan.<br>CS. Survey	1699	**Perception**:<br>37.3%, perceived CP a mere vendor.<br>44.6%, perceived CPs as an indispensable & effective part of the healthcare system.<br>**Satisfaction:**<br>71.4% of CPs available at the designated hours.<br>38.5% of CPs offer counseling without asking.<br>38.4% of CPs enquire about the related health problems, or any other medication used.<br>40.1% of CP asked about compliance of the previously dispensed medication.<br>51.2% of CPs instruct about duration of drug administration.	71%<br>M-High
Al-Tannir et al., 2016<br>Riyadh City	To assess adult customers' satisfaction level with PC services.	Quan.<br>CS. Survey	500	**Perceptions**:<br>Lack in pharmacy services. Different perceptions towards PC services.<br>**Satisfaction:**<br>41 % of participants reported being satisfied with overall services<br>57.2% of CP available at the designated hours. 4.8%, CP introduce him/herself.<br>33.2% of CP asked about concomitant medication. 41.2% of CP asked about comorbid diseases.<br>85.6% of CP explained dosage regimen. 40.6%, CP ensured understanding of dosage.<br>24.6% of CP explained side effects. 59.7%, CP discussed medication fears.<br>56.2% of CP asked about health & medication. 53.4%, CP gave enough time for counselling.	71%<br>M-High
Alhaddad et al., 2018<br>Nationwide	To assess females' satisfaction towards service provided by male CPs.	Quan.<br>CS. Survey	822	**Satisfaction**:<br>35.3% were satisfied with male CPs' services, 14.2% were not while 50.5% were satisfied to a certain extent.<br>**Dissatisfactions:**<br>The most important problems when female go to the community pharmacy:<br>63%, embarrassing to discuss with male CPs. 14.9%, Crowded. 9.0%, lack trust on CPs.<br><br>The need of female customers for female CP was 60,80%.	79%<br>M-High
