# Supplementary material for: Multi-stakeholder perspective on community pharmacy services in Saudi Arabia: A systematic review and meta-analyses for 2010–2020
Source: Explor Res Clin Soc Pharm. 2025 Apr 28;18:100608. doi: 10.1016/j.rcsop.2025.100608 (PMC12099458; doi:10.1016/j.rcsop.2025.100608)
Supplement: Supplementary material 4 — Quality assessment. [file mmc4.pdf]

Supplemental material 4. Quality assessment.

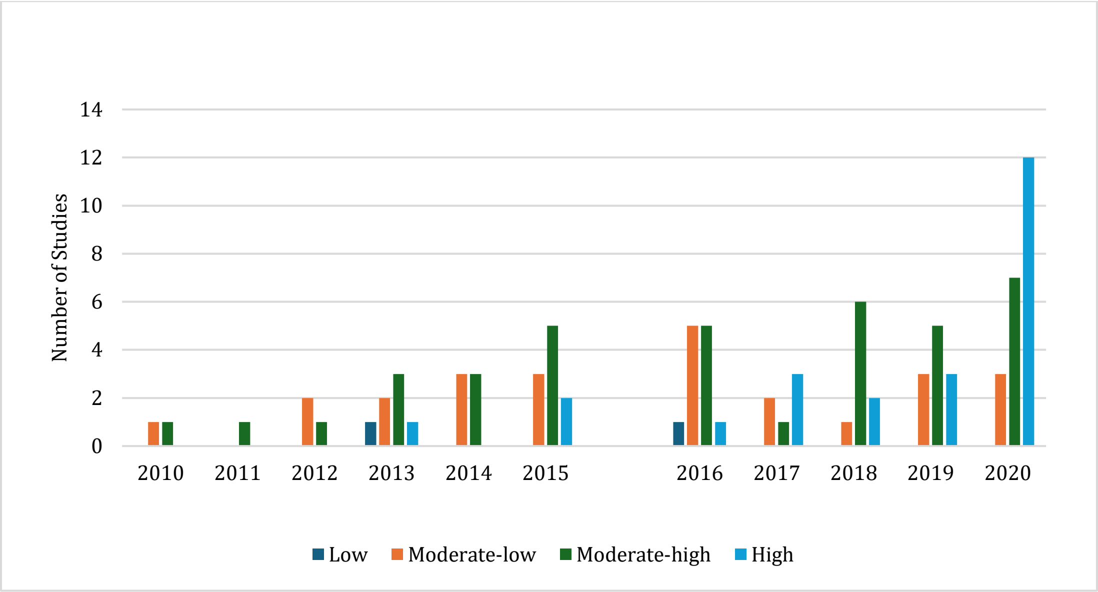

Figure 1. Methodological quality and publishing quantity of included studies 2010 - 2020 (n = 89)

Table 1. Quality assessment using MMAT.

References	Screening Questions		Quantitative					Qualitative					Mixed methods					MAAT	
	Clear research questions.	Data allow to address the research questions.	Sampling strategy.	Sample representative of the target population.	Are the measurements appropriate?	Is the risk of bias low?	Appropriate statistical analysis.	Qualitative approach appropriates to answer the research question.	Qualitative data collection methods adequate to address the research question.	The findings adequately derived from the data.	The interpretation of results sufficiently substantiated by data.	Coherence between data sources, collection, analysis & interpretation.	Rationale for using a mixed method.	Different components effectively integrated to answer the research question.	The outputs of the integration of mixed components adequately interpreted.	Addressed divergences and inconsistencies between two data.	components adhere to the criteria of each tradition of the methods involved.	Points / 7	Scores %
Al Hassan, 2010	1	1	0	1	1	0	0.5											4.5	64
Alkharfy, 2010	1	1	0.5	1	1	0	1											5.5	79
Bin Abdulhak et al., 2011	1	1	0.5	1	0.5	0	1											5	71
Al-Rukban & Khalil, 2012	1	0.5	0	1	1	0	1											4.5	64
Al-Arifi, 2012	1	0.5	0	1	0.5	1	1											5	71
Emeka et al., 2012	1	1						1	0	0	0.5	0.5						4	57
T. M. Khan & Ibrahim, 2013	1	1						1	0	0	1	1						5	71
Suleiman, 2013	1	1	0.5	1	1	1	1											6.5	93
Al-Mohamadi et al., 2013	1	1	0	1	0	0	1											4	57
T. M. Khan, 2013	1	1	0	1	1	0	1											5	71
Al-Hazmi & IL, 2013	1	1	0	1	0	0	0.5											3.5	50
T. M. Khan & Azhar, 2013	1	1	0	1	0.5	0	1											4.5	64
Al-Arifi, 2013	1	1	0	1	1	0	1											5	71
Alrwisan et al., 2014	1	1	0	1	1	0	1											5	71
Al-Arifi, 2014b	1	1	0	1	1	0	1											5	71
Al-rukban & Rizvi, 2014	1	0.5	0	1	0.5	0	1											4	57
Bawazir, 2014	1	1	0	1	0.5	0	1											4.5	64
M. A. Mahmoud et al., 2014	1	1	0	1	0.5	0	1											4.5	64
Al-Arifi, 2014a	1	1	0	1	1	0	1											5	71
Alaqeel & Abanmy, 2015	1	1	0	1	1	0	1											5	71
Suleiman, 2015	1	1	1	1	1	0	0.5											5.5	79
Alqahtani et al., 2015	1	1	0	1	0	0	1											4	57
Adnan et al., 2015	1	0.5	0	1	0.5	0	1											4	57
Rabba & Ain, 2015	1	1	0	1	1	0	1											5	71

Table 1. Quality assessment using MMAT (continued).

**References**	**Screening Questions**		**Quantitative**					**Qualitative**					**Mixed methods**					**MAAT**	
	Clear research questions.	Data allow to address the research questions.	Sampling strategy.	Sample representative of the target population.	Are the measurements appropriate?	Is the risk of bias low?	Appropriate statistical analysis.	Qualitative approach appropriates to answer the research question.	Qualitative data collection methods adequate to address the research question.	The findings adequately derived from the data.	The interpretation of results sufficiently substantiated by data.	Coherence between data sources, collection, analysis & interpretation.	Rationale for using a mixed method.	Different components effectively integrated to answer the research question.	The outputs of the integration of mixed components adequately interpreted.	Addressed divergences and inconsistencies between two data.	components adhere to the criteria of each tradition of the methods involved.	Points / 7	Scores %
Wajid et al., 2015	1	1	1	1	1	0	1											6	86
T. M. Khan & Emeka, 2015	1	1						1	0	0	1	1						5	71
Albadr & Khan, 2015	1	1						1	0	0	1	1						5	71
Aljadhey et al., 2015	1	1	0	1	1	1	1											6	86
Alotaibi & Abdelkarim, 2015	1	1	0	1	0.5	0	0.5											4	57
Al-Tannir et al., 2016	1	1	0	1	0	1	1											5	71
Alosaimi et al., 2016	1	1	0.5	1	1	1	1											6.5	93
Alotaibi et al., 2016	1	1	0	1	0.5	0	0.5											4	57
Kashour et al., 2016	1	1	0	1	1	0	1											5	71
Suleiman et al., 2016	1	1	0	1	0	0	1											4	57
Hadi et al., 2016	1	1	0	1	1	0	1											5	71
Al Akhali, 2016a	1	1	0	1	0	0	1											4	57
Alkhuzaee et al., 2016	1	1	0.5	1	1	0	1											5.5	79
Bahnassi, 2016	1	1	0	1	1	0	1											5	71
Alakhali, 2016b	1	1	0	1	0	0	1											4	57
Alahdal et al., 2016	1	1	0	1	0	0	0.5											3.5	50
Ahmed. N & AL-Wahibi, 2016	1	1	0	1	0	0	1											4	57
Alshammari et al., 2017	1	1	0	1	0	0	1											4	57
Al-Saleh et al., 2017	1	1	1	1	1	0	1											6	86
Alrabiah et al., 2017	1	1	0	1	1	0	1											5	71
Gillani et al., 2017	1	1	1	1	1	0	1											6	86
Zahrani et al., 2017	1	1	1	1	1	0	1											6	86
Mazhar et al., 2017	1	1						1	0	0	0	1						4	57
Kamel et al., 2018	1	1	0	1	1	0	1											5	71

Table 1. Quality assessment using MMAT (continued).

References	Screening Questions		Quantitative					Qualitative					Mixed methods					MAAT	
	Clear research questions.	Data allow to address the research questions.	Sampling strategy.	Sample representative of the target population.	Are the measurements appropriate?	Is the risk of bias low?	Appropriate statistical analysis.	Qualitative approach appropriates to answer the research question.	Qualitative data collection methods adequate to address the research question.	The findings adequately derived from the data.	The interpretation of results sufficiently substantiated by data.	Coherence between data sources, collection, analysis & interpretation.	Rationale for using a mixed method.	Different components effectively integrated to answer the research question.	The outputs of the integration of mixed components adequately interpreted.	Addressed divergences and inconsistencies between two data.	components adhere to the criteria of each tradition of the methods involved.	Points / 7	Scores %
M. Mahmoud et al., 2018	1	1						1	0	0.5	1	1						5.5	79
Al Aqeel & Hiligsmann, 2018	1	1											0	1	0	1	1	5	71
Alhomoud et al., 2018	1	1						1	0.5	0.5	1	1						6	86
Alhaddad et al., 2018	1	1	0	1	1	1	0.5											5.5	79
Alsayari et al., 2018	1	1	1	1	1	0	1											6	86
Alfadl et al., 2018	1	1	0	1	1	0	0.5											4.5	64
A. Mahmoud et al., 2018	1	1	0	1	1	0	1											5	71
Balkhi et al., 2018	1	1	0	1	1	0	1											5	71
Alhaddad, 2019	1	1	0	1	1	1	1											6	86
Alrabiah et al., 2019	1	1	1	1	0.5	0	1											5.5	79
Khojah, 2019a	1	1	0.5	1	0.5	0	1											5	71
Ghosn et al., 2019	1	1	0.5	1	0.5	0	1											5	71
Al Juffali et al., 2019a	1	1						1	1	1	1	1						7	100
Al Juffali et al., 2019b	1	1	1	0.5	1	1	1											6.5	93
Khojah, 2019b	1	1	0	1	0	0	1											4	57
Khojah & Abdalla, 2019	1	1	0	1	0	0	1											4	57
Ahmed & Khan, 2019	1	1	0	1	1	0	0.5											4.5	64
Alrefaei et al., 2019	1	1	0	1	0.5	1	1											5.5	79
Alhossan et al., 2019	1	1	0	1	1	0	1											5	71
Alrasheedy et al., 2020	1	1	0.5	1	0.5	0	1											5	71
Al-Tannir et al., 2020	1	1	0	1	1	0	0.5											4.5	64
Alrukban et al., 2020	1	1	0.5	1	1	1	1											6.5	93
S. M. Alshahrani, 2020	1	1	0.5	1	1	1	1											6.5	93
Aloudah et al., 2020	1	1											1	0.5	0.5	0.5	0.5	5	71

Table 1. Quality assessment using MMAT (continued).

References	Screening Questions		Quantitative					Qualitative					Mixed methods					MAAT	
	Clear research questions.	Data allow to address the research questions.	Sampling strategy.	Sample representative of the target population.	Are the measurements appropriate?	Is the risk of bias low?	Appropriate statistical analysis.	Qualitative approach appropriates to answer the research question.	Qualitative data collection methods adequate to address the research question.	The findings adequately derived from the data.	The interpretation of results sufficiently substantiated by data.	Coherence between data sources, collection, analysis & interpretation.	Rationale for using a mixed method.	Different components effectively integrated to answer the research question.	The outputs of the integration of mixed components adequately interpreted.	Addressed divergences and inconsistencies between two data.	components adhere to the criteria of each tradition of the methods involved.	Points / 7	Scores %
Mobrad et al., 2020	1	1	0	1	1	0	1											5	71
Malebari et al., 2020	1	1	0	1	1	1	1											6	86
Al Qarni et al., 2020	1	1	0.5	1	0.5	0	1											5	71
A. Alshahrani, 2020a	1	0.5	0	1	1	1	1											5.5	79
Kurban et al., 2020	1	0.5	0	1	0.5	0	1											4	57
Ali et al., 2020	1	1	0	1	1	1	1											6	86
A. Alshahrani, 2020b	1	1	1	1	0	1	1											6	86
Khojah, 2020	1	1	0	1	0.5	0	0.5											4	57
Almubark et al., 2020	1	1	0.5	1	1	1	1											6.5	93
Ansari et al., 2020	1	1	1	1	1	1	1											7	100
K. A. Khan et al., 2020	1	1	0	1	0.5	1	0.5											5	71
AlShayban et al., 2020	1	1	1	1	1	1	1											7	100
Almansour et al., 2020	1	1						1	0.5	0.5	1	1						6	86
Al Aloola et al., 2020	1	1						1	0.5	0.5	1	1						6	86
Alzayer et al., 2020	1	0.5						1	0.5	0.5	1	0.5						5	71
Y. Khan et al., 2020	1	1	0.5	1	1	1	1											6.5	93
Rasheed et al., 2020	1	1						1	0.5	0.5	1	1						6	86

The 'Can't tell' response category means that the paper do not report appropriate information to answer, 'Yes' or 'No', or that report unclear information (Hong QN. et al, 2018).

1	**Yes**
0.5	**Can't Tell***
0	**No**

Quality

≤ 50%	51% - 65%	66% - 79%	≥ 80%
Weak	Moderate-low	Moderate-high	High
