# Supplementary material for: Multi-stakeholder perspective on community pharmacy services in Saudi Arabia: A systematic review and meta-analyses for 2010–2020
Source: Explor Res Clin Soc Pharm. 2025 Apr 28;18:100608. doi: 10.1016/j.rcsop.2025.100608 (PMC12099458; doi:10.1016/j.rcsop.2025.100608)
Supplement: Supplementary material 5 — Forest plots of meta-analysis. [file mmc5.pdf]

Supplemental material 5. Forest plots of meta-analyses

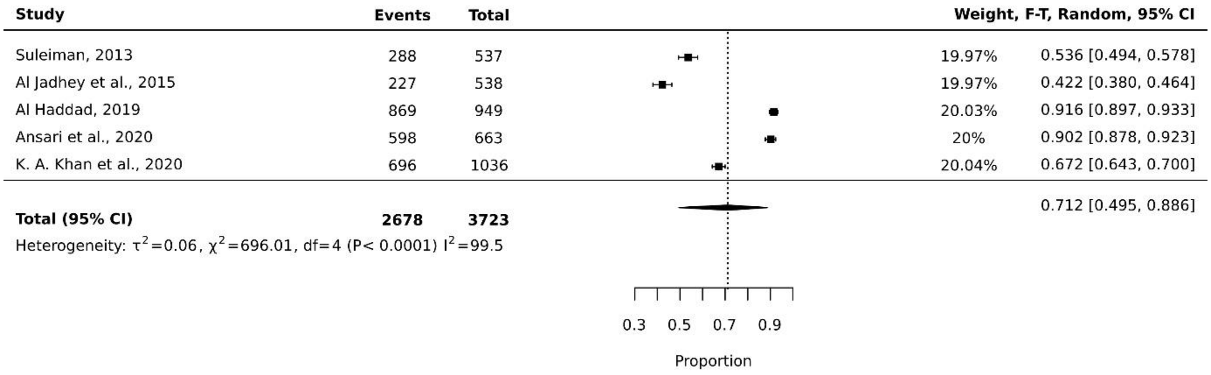

Figure 1. The proportion of self-medication for minor symptoms by public.

Dispensing POMs without prescription

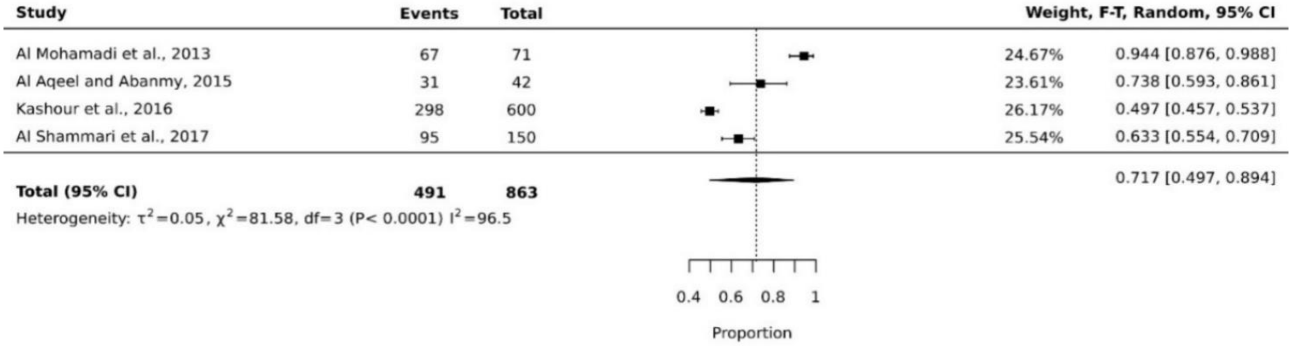

Dispensing antibiotics without prescription (before it was outlawed)

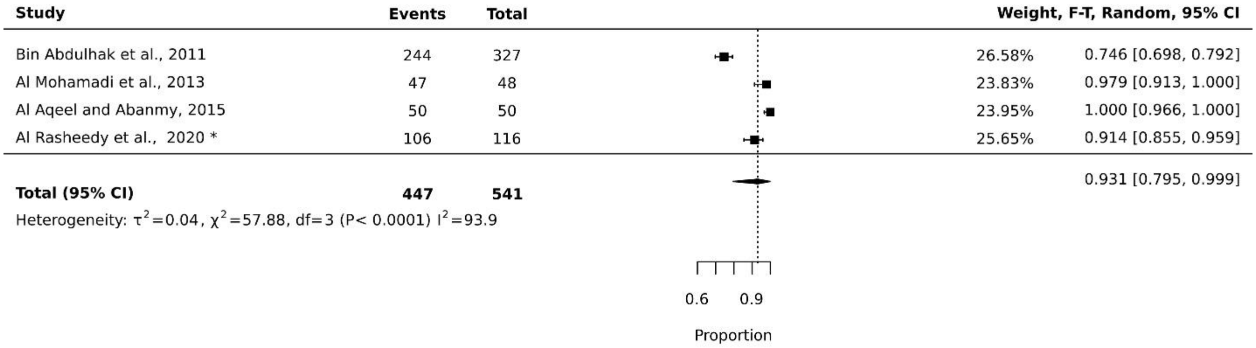

Dispensing Antibiotic without prescription (after it was outlawed)

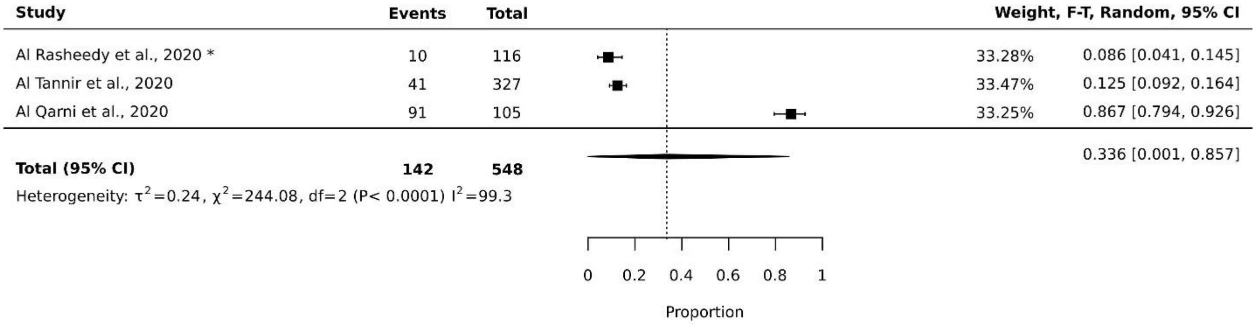

- Al Rasheedy et al., 2020 had two phases: a pre-illegality phase in 2017, and a post-illegality phase in 2018

Figure 2. The proportion of dispensing POMs and antibiotics without prescription.

If not dispensed, the patient can obtain it from any CP

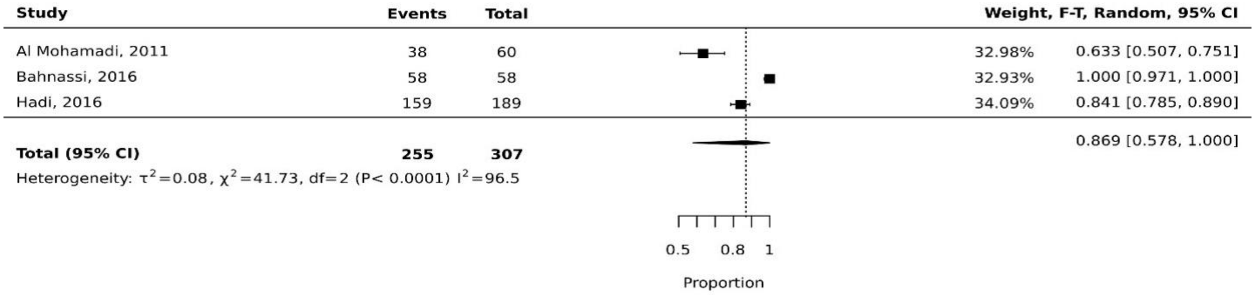

Patient’ socioeconomic status

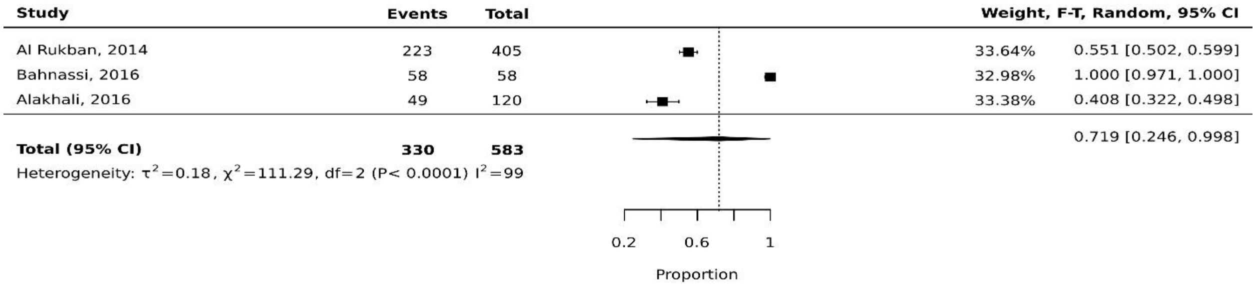

Simple symptoms

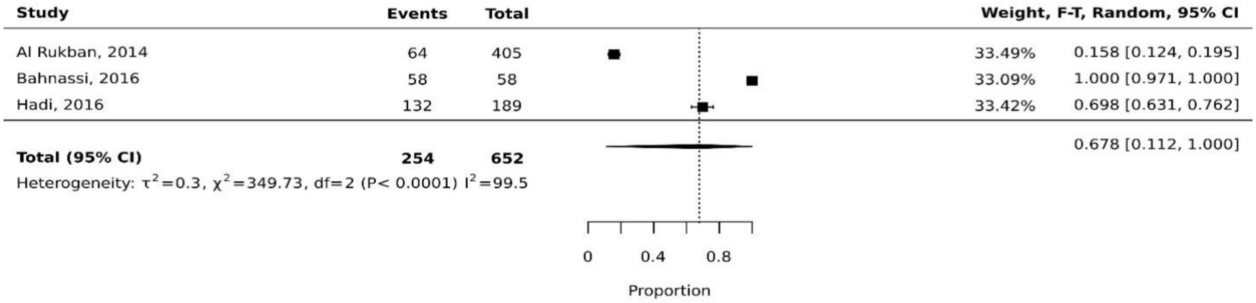

Pharmacists’ confidence/ knowledge

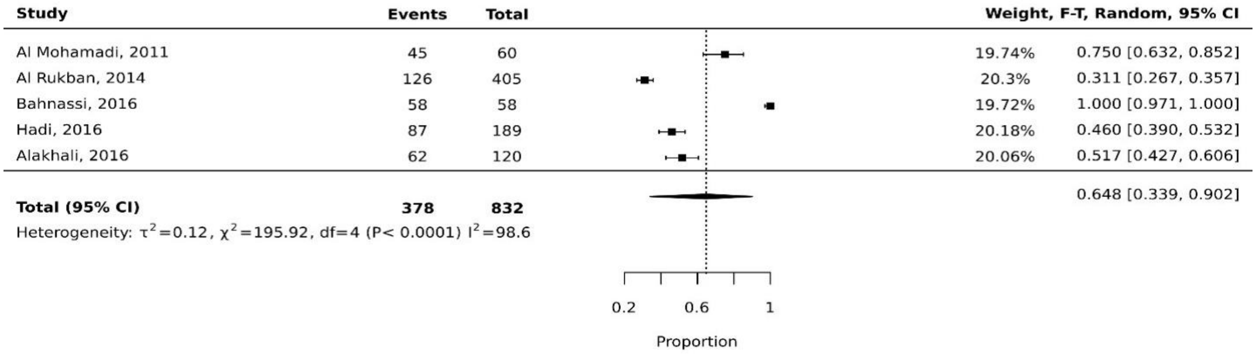

Difficulties in reaching the clinic

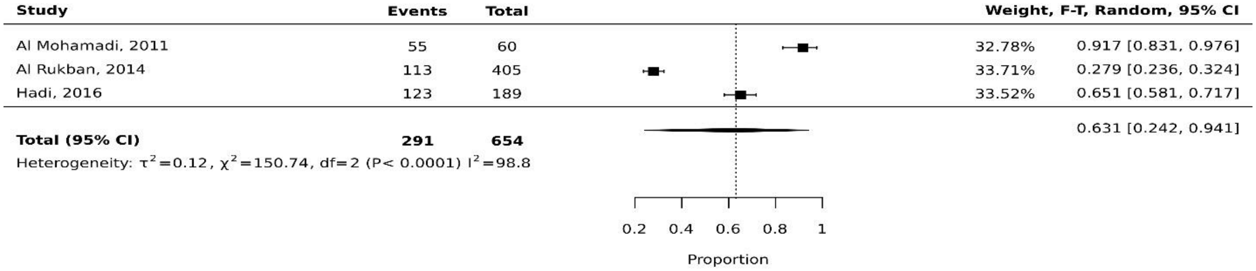

Figure 3. The reasons for community pharmacists dispensing POMs, including antibiotics

Asking about associated symptoms

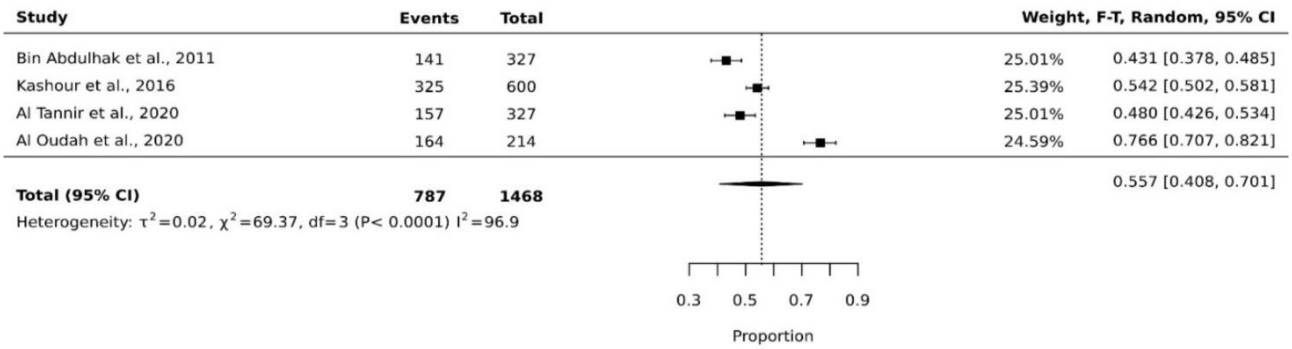

Asking about concomitant drugs/comorbidities

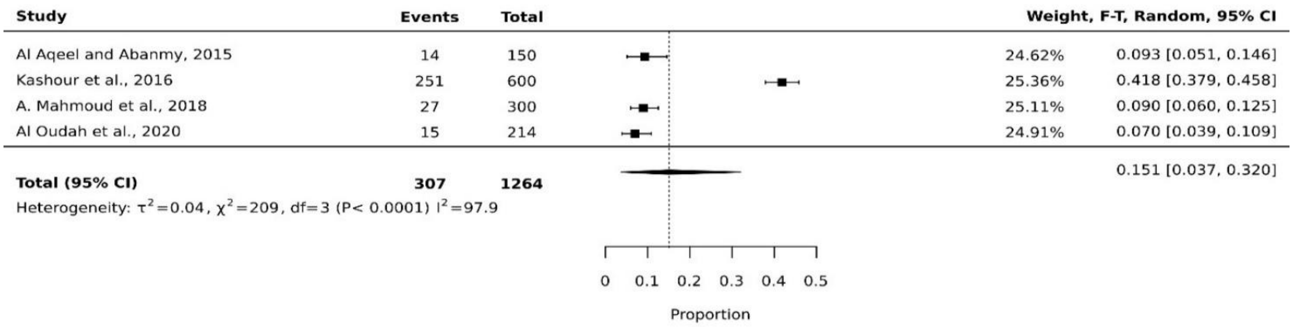

Asking if the patient had taken this medicine before

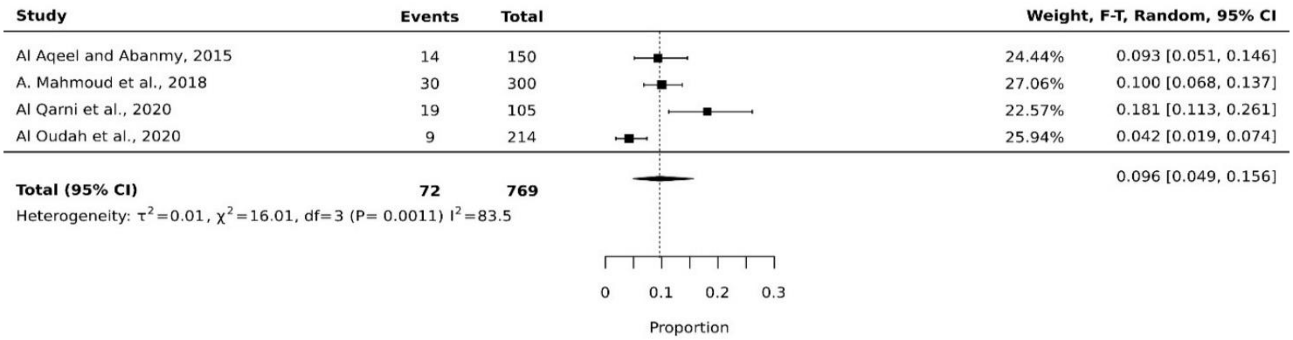

Figure 4 The proportion of patient counselling attributes practiced by community pharmacists

Asking about drug allergies

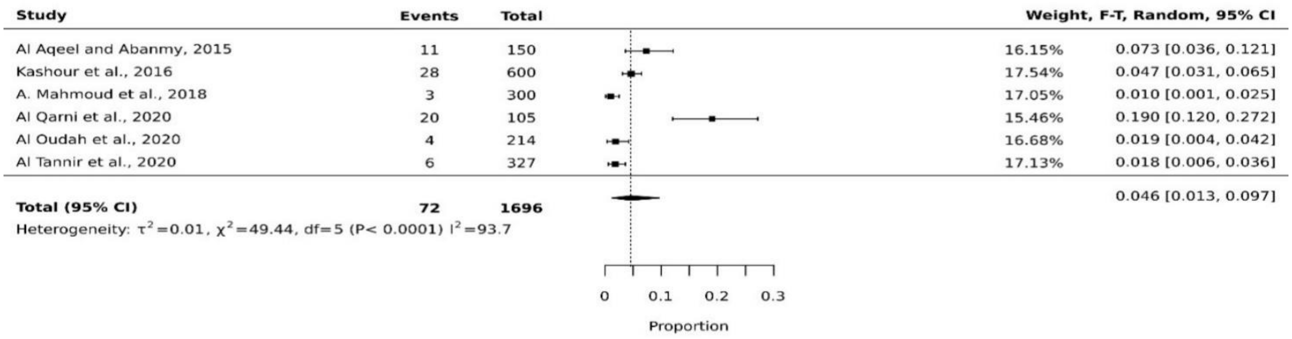

Asking about pregnancy status

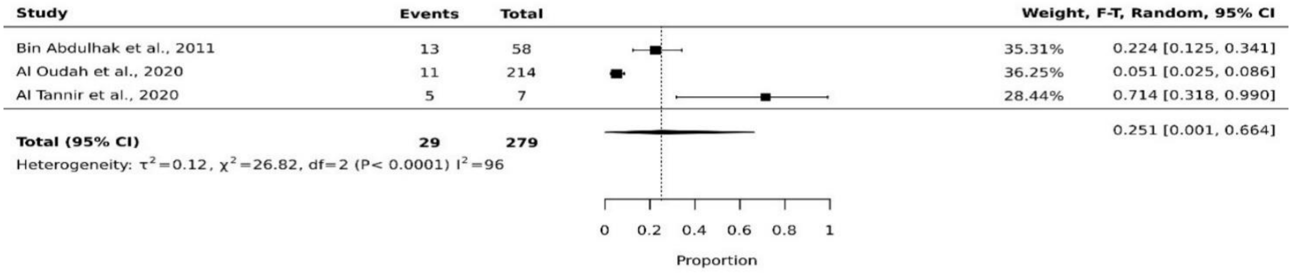

Providing duration of use

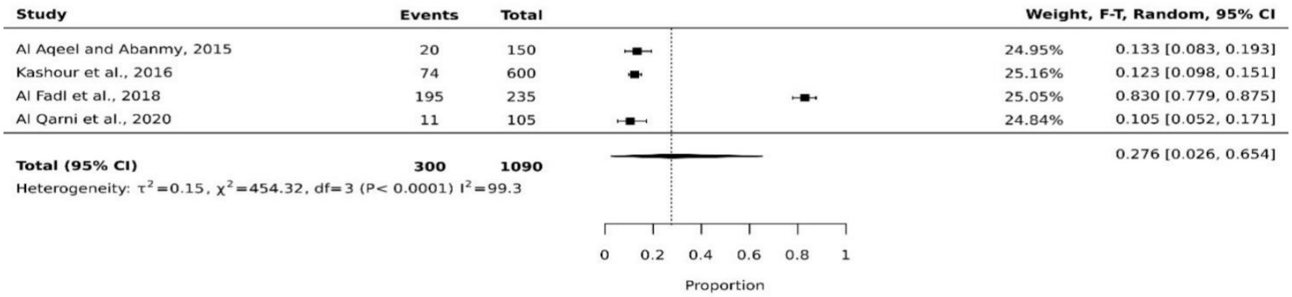

Demonstrating correct use of metered dose inhaler steps

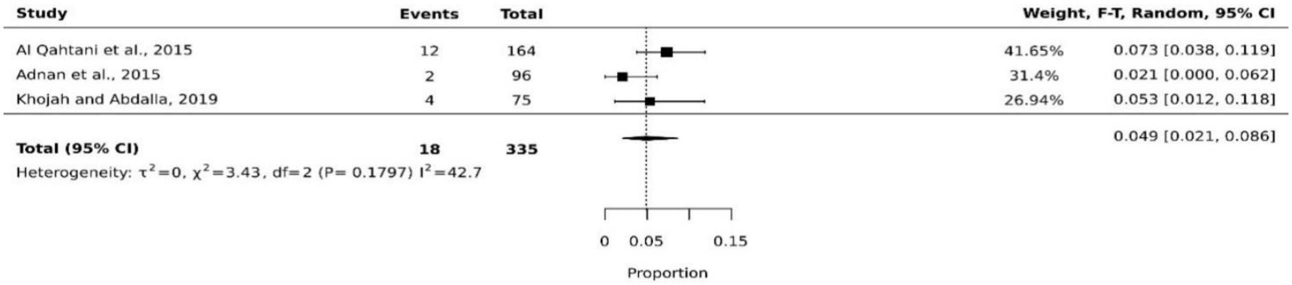

Figure 4. The proportion of patient counselling attributes practiced by community pharmacists (continued)

Lack of time

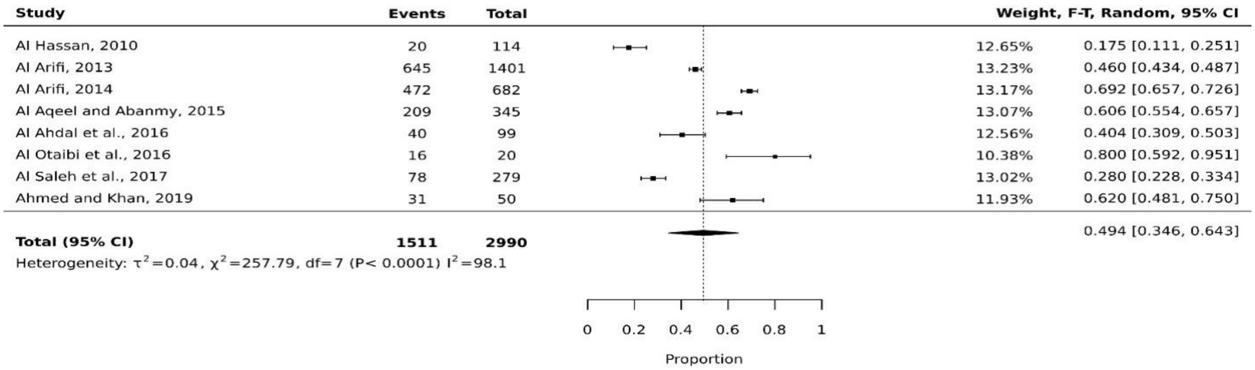

Lack of reliable sources /information

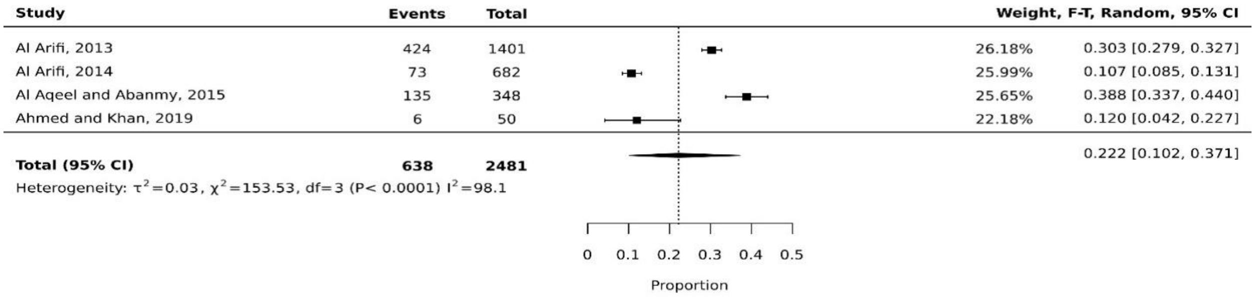

Patient culture/not interested

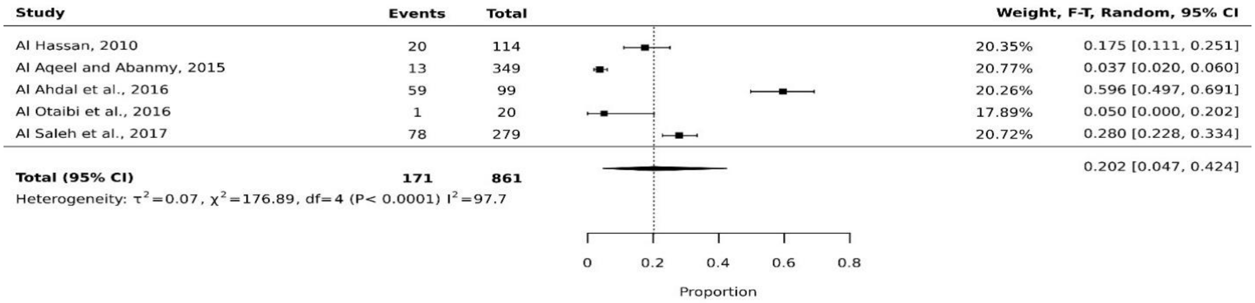

Lack of pharmacists’ confidence/ knowledge

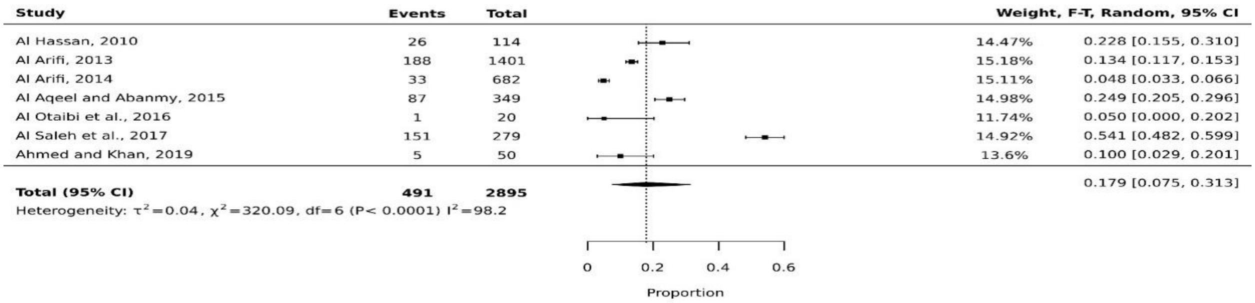

Community pharmacists are not interested

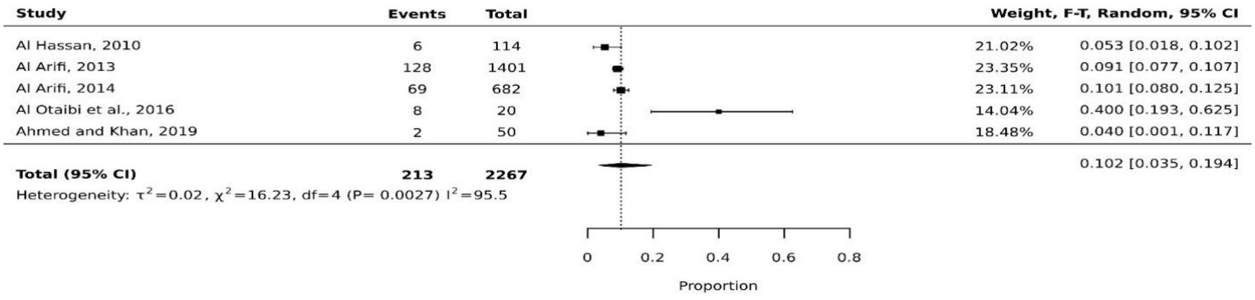

Figure 5. The barriers to patient counselling by community pharmacists

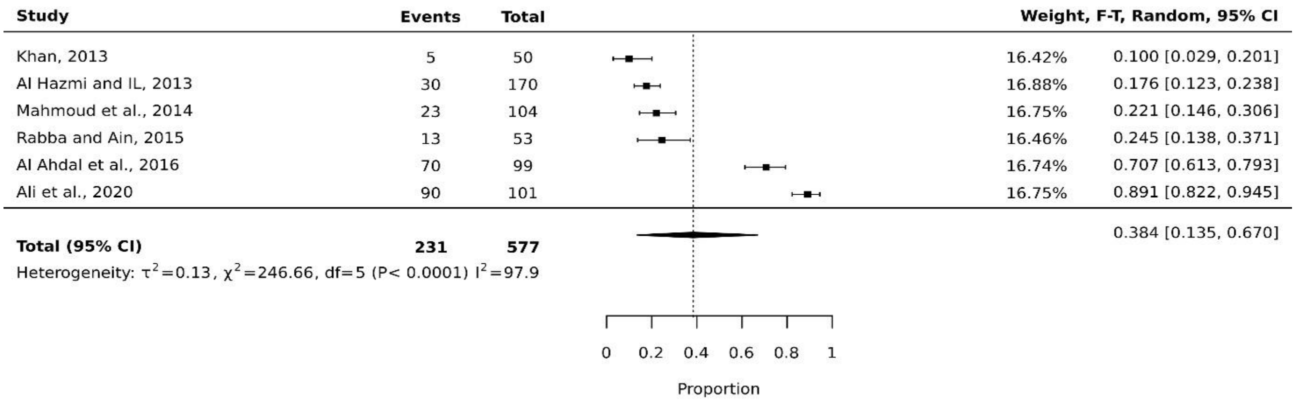

Figure 6. Community pharmacists' familiarity with ADR reporting system

Reporting forms are not available

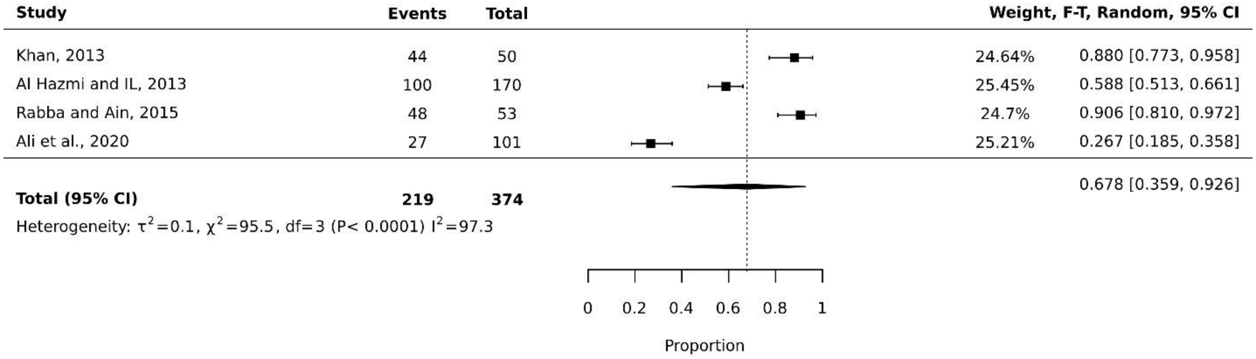

No motivation

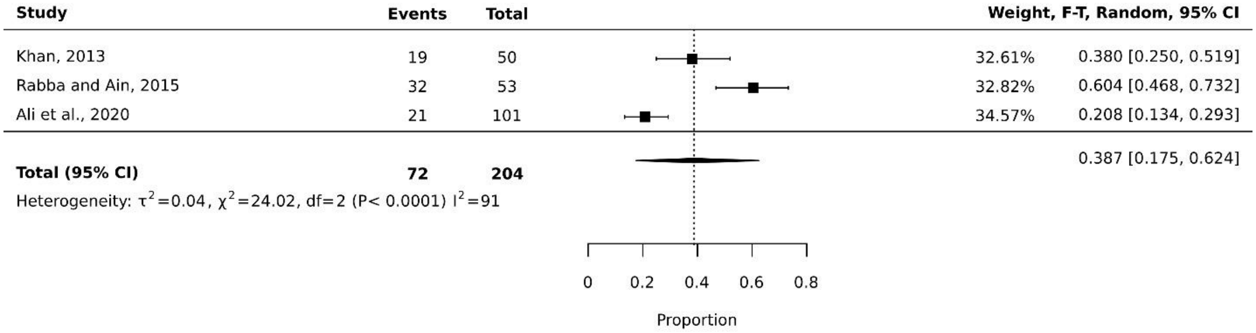

I am not sure if it’s ADRs

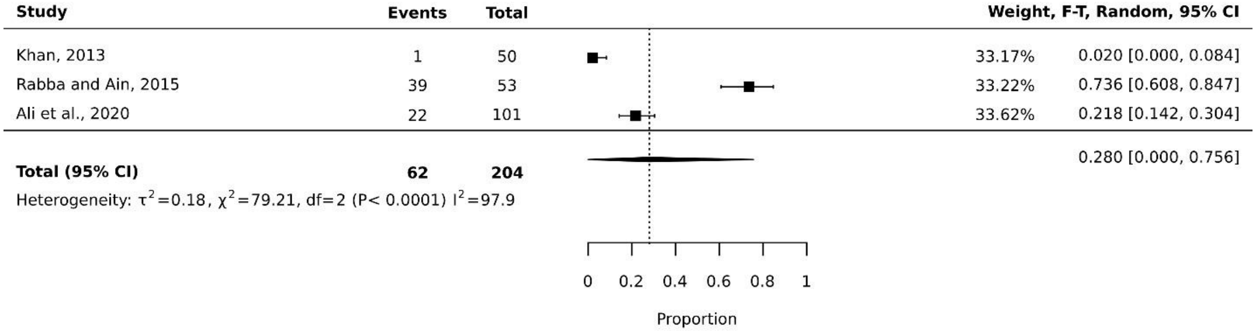

Reporting is time consuming

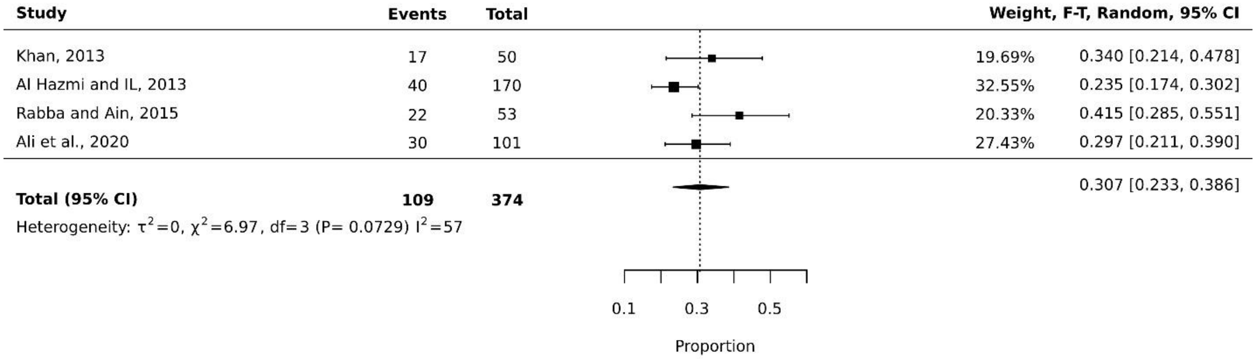

Figure 7. The barriers to ADRs reporting system by community pharmacists

Fear of legal liability

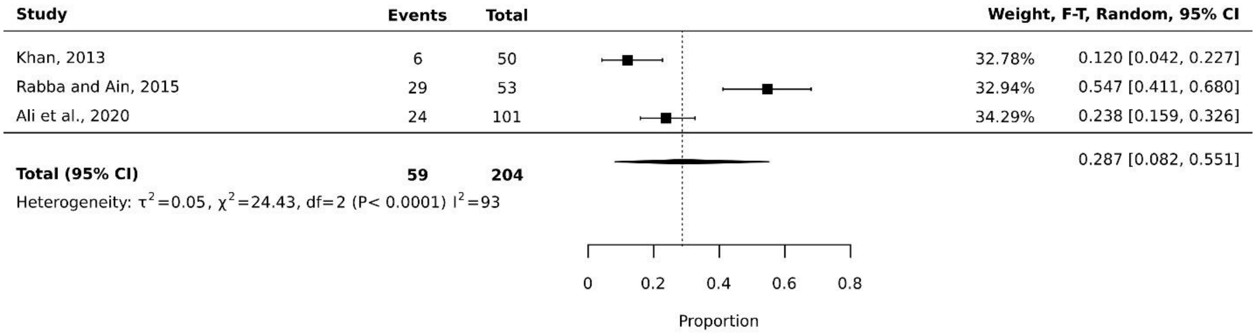

Forms are too complicated

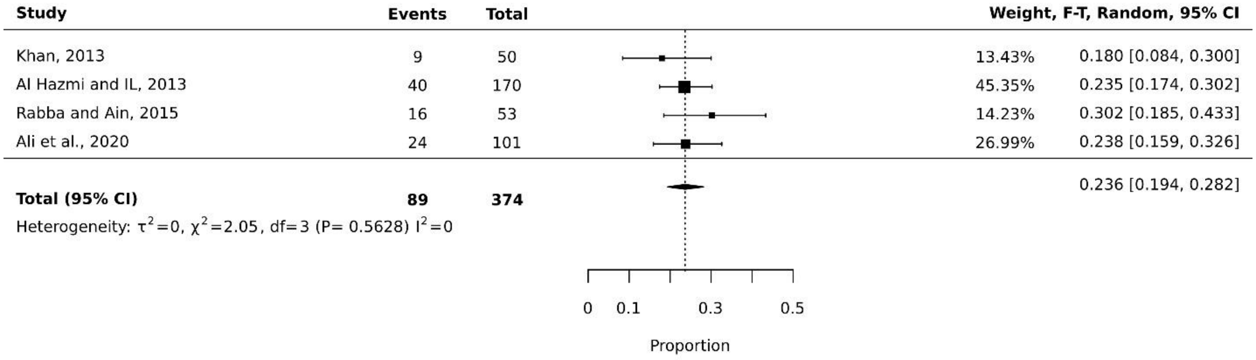

Insufficient clinical knowledge

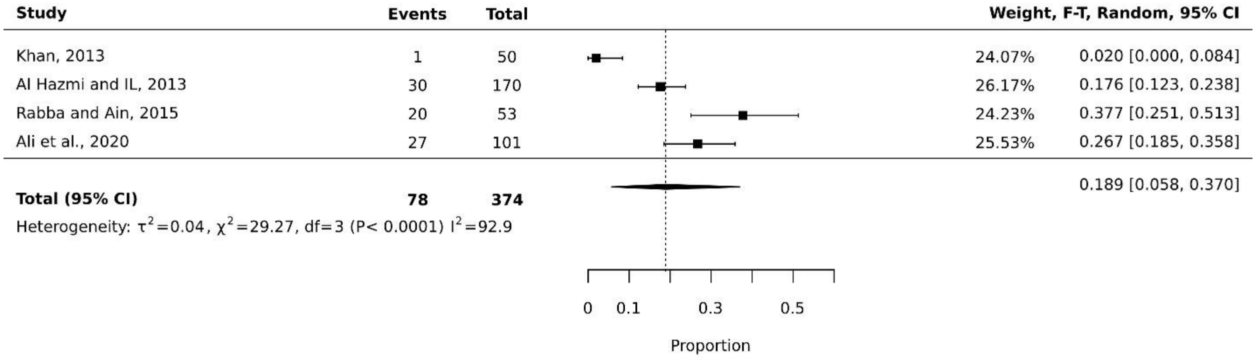

Figure 7. The barriers to ADRs reporting system by community pharmacists (continued)
